# Private healthcare provider experiences with social health insurance schemes: Findings from a qualitative study in Ghana and Kenya

**DOI:** 10.1371/journal.pone.0192973

**Published:** 2018-02-22

**Authors:** Maia Sieverding, Cynthia Onyango, Lauren Suchman

**Affiliations:** 1 Global Health Sciences, University of California San Francisco, San Francisco, California, United States of America; 2 Innovations for Poverty Action, Nairobi, Kenya; Universitas Sebelas Maret Fakultas Kedokteran, INDONESIA

## Abstract

**Background:**

Incorporating private healthcare providers into social health insurance schemes is an important means towards achieving universal health coverage in low and middle income countries. However, little research has been conducted about why private providers choose to participate in social health insurance systems in such contexts, or their experiences with these systems. We explored private providers’ perceptions of and experiences with participation in two different social health insurance schemes in Sub-Saharan Africa—the National Health Insurance Scheme (NHIS) in Ghana and the National Hospital Insurance Fund (NHIF) in Kenya.

**Methods:**

In-depth interviews were held with providers working at 79 facilities of varying sizes in three regions of Kenya (N = 52) and three regions of Ghana (N = 27). Most providers were members of a social franchise network. Interviews covered providers’ reasons for (non) enrollment in the health insurance system, their experiences with the accreditation process, and benefits and challenges with the system. Interviews were coded in Atlas.ti using an open coding approach and analyzed thematically.

**Results:**

Most providers in Ghana were NHIS-accredited and perceived accreditation to be essential to their businesses, despite challenges they encountered due to long delays in claims reimbursement. In Kenya, fewer than half of providers were NHIF-accredited and several said that their clientele were not NHIF enrolled. Understanding of how the NHIF functioned was generally low. The lengthy and cumbersome accreditation process also emerged as a major barrier to providers’ participation in the NHIF in Kenya, but the NHIS accreditation process was not a major concern for providers in Ghana.

**Conclusions:**

In expanding social health insurance, coordinated efforts are needed to increase coverage rates among underserved populations while also accrediting the private providers who serve those populations. Market pressure was a key force driving providers to gain and maintain accreditation in both countries. Developing mechanisms to engage private providers as stakeholders in social health insurance schemes is important to incentivizing their participation and addressing their concerns.

## 1. Introduction

Growing international commitment to Universal Health Coverage (UHC) has brought increased attention to the potential role of social health insurance (SHI) in achieving population access to affordable healthcare. The World Health Organization defines UHC as “…access to key promotive, preventive, curative and rehabilitative health interventions for all at an affordable cost…” [1]. This is achieved through financial risk pooling, in which the majority of a population pays into a central healthcare fund. Although there are a number of options for health financing through risk pooling, countries pursuing UHC have generally employed one of two types of systems: 1) a National Health Service, in which general tax revenue is used to pay a network of public and private providers; or 2) Social Health Insurance schemes, which require compulsory membership from the entire population and are paid through contributions from workers’ salaries [1–3].

SHI systems in many low- and middle-income countries (LMICs), including in Sub-Saharan Africa, are nascent and face challenges in achieving broad population coverage. These challenges include extending coverage to the large sector of informal workers present in many LMICs [4], low re-enrollment rates among clients [5], mistrust of the public health system [6] and government transparency in managing SHI systems [7], and difficulties securing sustainable government funding [8,9]. Further, there is potential for the push towards UHC to increase the health coverage gap between rich and poor if poor populations do not have adequate access to health insurance schemes [10] or cannot afford them when they do [11]. A “pro-poor” stance is therefore important when developing SHI schemes [12], particularly in countries where access to quality healthcare is already unequal [13].

The private sector delivers a large proportion of healthcare services in many Sub-Saharan African countries, and often fills gaps left in serving poor populations [14]. Effective private sector involvement in the health system is therefore important to achieving UHC [15], including private sector involvement in SHI schemes. However, much of the literature on SHI schemes in LMICs focuses on public provision of healthcare [16], or does not differentiate between public and private sector providers [17,18]. We therefore know very little about private providers’ experiences with social health insurance in Sub-Saharan Africa or other LMICs. To the best of our knowledge, no previous studies have focused on private providers’ perspectives on an SHI system in a Sub-Saharan African context, or their reasons for participating in or opting out of such a system.

In this paper, we examine private providers’ experiences with two SHI systems in Sub-Saharan Africa that are at very different stages of development: the National Health Insurance Scheme (NHIS) in Ghana and the National Hospital Insurance Fund (NHIF) in Kenya. These two cases pose an interesting comparison because of the different structures and current coverage levels of the SHI systems, which are described in more detail below. At the same time, assessments of the private healthcare sectors in both Kenya and Ghana indicate that private providers are an important source of care across wealth quintiles, making the integration of this sector into the SHI system important for both countries [19,20].

We also chose these two countries because this study was conducted as part of the evaluation of a social franchising intervention in Ghana and Kenya (see Methods section for more detail). Social franchises are networks of private sector facilities that are contracted, typically by a Non-Governmental Organization, to provide standard services under a common brand [21,22]. Social franchising is well established in Kenya, where it has been in existence since 2000 and several networks are in operation, with a combined membership of over 1,000 facilities in 2015 [22,23]. In contrast, there is only one large-scale franchise network in Ghana—the BlueStar network included in this study—with a membership of 189 facilities in 2015 [22]. Although discussions about the role of social franchising in UHC are still emerging, as an organizing mechanism for the private sector social franchises hold the potential to be a link between private providers and SHI systems [24].

Effectively engaging the private sector in SHI in Ghana, Kenya and other Sub-Saharan African contexts will require policymakers to consider the incentives, motivations, and business operations of private providers in the scheme design. In this paper, we aim to address the gap in the literature on private providers’ involvement in SHI schemes by examining the views of providers in Kenya and Ghana on participation in the NHIF and NHIS, and their experiences with these schemes. We also aim to understand how private providers make decisions about whether or not to apply for accreditation with SHI systems, in order to inform programmatic efforts to engage private providers in the expansion of SHI.

### 1.1 The National Health Insurance Scheme in Ghana

Ghana’s National Health Insurance Scheme was established in 2003 and fully implemented in 2005, at which point members could access benefits. Membership is legally mandatory for the whole population, but in practice enrollment is voluntary outside the formal sector. Several population groups are exempt from membership fees, including those over age 70, those under age 18 where both parents are members, and indigents [8]. Although exact figures are debated, coverage rates appear to have grown substantially since the scheme’s establishment, from an estimated 7 percent of the population in 2006 [8] to 38 percent in 2013 [25].

NHIS aims to provide a single system of coverage for the entire population [9] and the benefits package is the same for all population groups [26]. All providers participating in NHIS must offer a minimum package of services. It is estimated that NHIS benefits cover 95 percent of health conditions in Ghana, including inpatient and outpatient care, emergency services, and medicines [8,26]. Any provider may participate in NHIS once accredited; accredited facilities range from chemist shops to hospitals, and 40 percent are private sector providers [25]. Participating providers send claims to the NHIS for reimbursement, and payments should be made within four weeks of claims submission [8].

A substantial literature has emerged on the client-side dynamics of the NHIS, including predictors of enrollment and reenrollment [11,27–31], impacts on service utilization and out-of-pocket health expenditures [29,32] and client satisfaction with NHIS benefits [11,31]. In contrast, existing literature on provider experiences with and perceptions of the NHIS relies on very small samples [33,34] and primarily on interviews with public sector providers [5,16]. These studies have raised concerns that providers may treat clients with NHIS coverage unfavorably compared to those who pay in cash and may charge informal service fees to NHIS clients, practices that are likely linked to the pervasive problem of delayed reimbursements from NHIS [5,16,33–35]. In the largest of the qualitative studies on provider perspectives on NHIS that we identified, Dalinjong et al. (2012) interviewed 15 public sector providers in the Upper East region, where providers complained that NHIS reimbursements were delayed up to six months, leading to challenges with purchasing medicines [16]. How the introduction of NHIS and the widespread reimbursement delays have affected private sector providers that are for-profit and whose staff (unlike public facilities) are not paid by the government has not been explored, and was noted by the Dalinjong et al. study as a specific area requiring further research [16].

### 1.2 The National Hospital Insurance Fund in Kenya

The National Hospital Insurance Fund in Kenya contrasts with the NHIS in Ghana in that it is not a broad-based social health insurance scheme [7]. The NHIF was established in 1966 as mandatory health insurance covering inpatient services for formal sector employees and civil servants. In 1972, voluntary membership in NHIF was opened to the informal sector, or anyone who is not a formal sector employee [7]. Informal sector enrollees pay a flat-rate monthly contribution to cover a nuclear family, but as of 2005 only about half a million people were estimated to be covered through this mechanism ([36] cited in [37]). As of 2011, an estimated 20 percent of Kenya’s population was covered by the NHIF [9].

Although various efforts have been made since the early 2000s to transform the NHIF into an SHI scheme for Kenya, these have thus far been unsuccessful [7,9]. At the same time, NHIF has been introducing changes designed to increase membership and expand the benefits package. Starting in 2004, the previously limited benefits package was expanded to include the majority of inpatient care [37]. NHIF also recently rolled out an outpatient scheme that covers preventative and curative services, including medicines and chronic illness management [38].

NHIF members can access inpatient services at government facilities, as well as private for- or non-profit facilities that are accredited by NHIF [39]. The creation of the outpatient scheme has for the first time opened NHIF accreditation possibilities for facilities that do not offer inpatient services. The provider payment system is also different for the two schemes. For inpatient services, facilities are reimbursed by NHIF after services have been provided [37] and receive a flat daily fee that depends on the facility’s accreditation level, which is tied to the range of services offered [6]. The outpatient scheme functions on a capitation basis, in which facilities are paid a flat monthly fee for each NHIF user that has selected the facility as their primary healthcare facility [40,41]. The NHIF has been less well studied than the NHIS in Ghana, and as noted above, to the best of our knowledge no previous studies have examined providers’ perspectives on or experiences with the NHIF. This is a critical gap in the knowledge base as Kenya moves to expand NHIF coverage both in terms of population and services offered.

## 2. Methods

The data for this paper were collected as part of the qualitative evaluation of the African Health Markets for Equity (AHME) program in Ghana and Kenya. AHME works through social franchises to provide a package of quality improvement and financing interventions. One of the objectives of AHME is to improve the integration of social franchise facilities into the NHIS in Ghana and NHIF in Kenya, in part by providing support with gaining accreditation in the respective insurance scheme. The data presented in this paper were collected prior to the start of targeted AHME assistance on accreditation or other aspects of facilities’ interactions with the SHI systems in their respective countries.

The qualitative dataset that we analyze consists of in-depth interviews with private sector healthcare providers at facilities that were members of one of the AHME partner social franchises, as well as facilities that had been approached to join the franchise network but declined. Several measures were undertaken to increase the validity and reliability of the study both within each country and across the two countries during the sample selection, data collection and analysis phases. These measures are detailed in each of the relevant sections of the study methods.

### 2.1 Sample selection

Our primary population of facilities was selected from the members of three social franchise networks: the BlueStar network in Ghana and the Amua and Tunza networks in Kenya. BlueStar and Amua are affiliated with Marie Stopes International, whereas Tunza is affiliated with Population Services International (see [22] for more details on these networks). Our initial sampling strategy involved the selection of facilities from two groups: (1) facilities that had joined a franchise during the AHME implementation period, and (2) facilities that had been approached to join a franchise during the implementation period but had declined. The latter group of facilities was included to better understand how the decision to join a franchise may relate to other facility circumstances, including NHIS/NHIF accreditation status, and serves as a comparison group to the main sample. To conduct the sample selection, each franchise network provided the study team with lists of their existing franchised facilities, as well as facilities that had recently been approached for franchising but declined to join the network. Data provided on franchised facilities also included year of joining the franchise, the facility’s NHIS or NHIF accreditation status, and its participation in the different components of the AHME interventions (including which franchise network was joined and participation in other financing and quality improvement programs).

**Fig 1:**
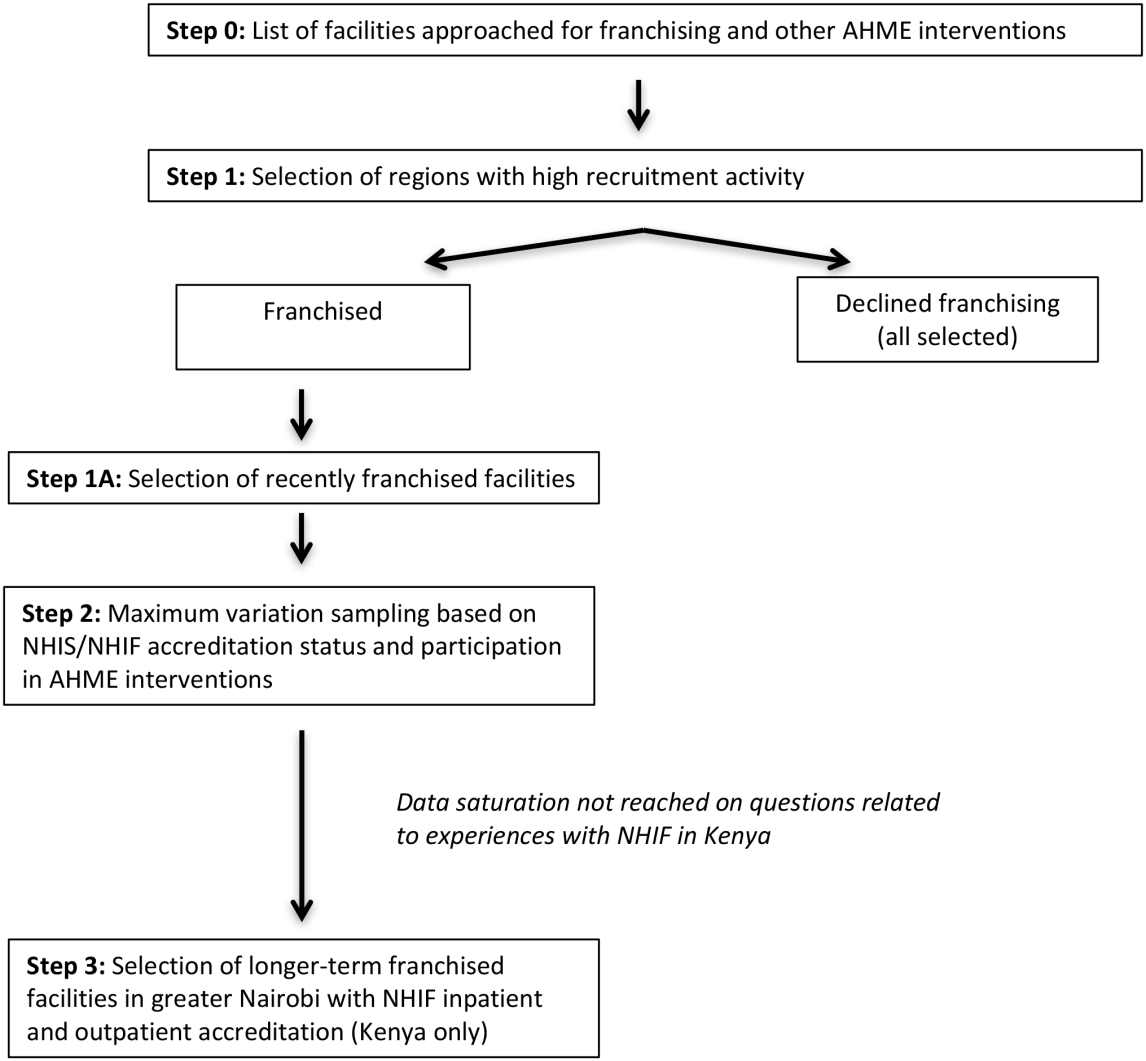
Process for selecting the qualitative provider sample.

In order to better understand the range of facilities’ experiences with the AHME interventions and potential needs with regards to participation in the national SHI scheme, we adopted a two-stage purposive sampling strategy (Fig 1) [42]. The first stage followed a criterion sampling method, in which we narrowed our sampling frame to three regions in each country where the AHME partners had conducted substantial recruitment. The selected regions were the Nairobi, Rift Valley and Eastern regions in Kenya, and the Greater Accra, Eastern and Western regions in Ghana. Due to the smaller number of facilities that declined franchising, we selected all of the facilities meeting this criterion in the three regions for interview. Among franchised facilities, we further narrowed our frame to facilities that had joined one of the networks in the past two years, to explore facilities’ reasons for joining the social franchise with less recall bias than might be introduced with facilities that had been part of the network for longer.

Within each region, we then selected franchised facilities using a maximum variation sampling method based on NHIS/NHIF accreditation status and participation in the different AHME intervention components. We selected a larger sample of facilities in Kenya than in Ghana because two franchise networks were included in Kenya. The response rate among the selected sample of facilities was higher among those who had agreed to join the franchise than among those who had declined. Of the 40 franchised facilities originally selected in Kenya, four declined to participate or were determined ineligible either because they were found not to be members of a franchise (N = 1), the owner had changed (N = 2), or the owner was not interested in participating (N = 1). Of the 26 franchised facilities selected in Ghana, 2 refused to participate due to lack of interest. Of the 10 facilities that declined franchising selected for interview in Kenya, 7 agreed to participate in an interview, and of the 5 facilities selected in Ghana, 3 agreed to an interview. The remaining facilities refused to participate in the study.

In order to increase the reliability of the results across Ghana and Kenya, we followed the same sampling approach in both countries. However, after conducting the initially planned interviews in Kenya, the research team determined that data saturation had not been reached on questions related to experiences with NHIF because some respondents had low levels of knowledge of NHIF and could not answer questions about the scheme functioning. We therefore added a third sample category in Kenya only; using criterion sampling, we approached 14 additional facilities in the Nairobi area that joined the franchise prior to AHME implementation, but had both NHIF inpatient and outpatient accreditation for interview. Nine of these facilities agreed to participate in the study, whereas the others could not be reached or said they were too busy for an interview.

### 2.2 Data collection

Interviews were conducted in Kenya in July 2015, and in Ghana in October and November 2015. A team of four local field staff in Kenya and three local field staff in Ghana with previous experience in qualitative data collection conducted the interviews. In each country, the same three-day training was held to ensure consistency among interviewers in their understanding and application of the interview guide, and thus to increase the validity and reliability of the data across interviewers and countries. This training, conducted by the authors, covered the study objectives, IRB-approved study protocols, a discussion of the purpose of each question in the interview guide, and interviewing techniques. Among other topics, the sessions on interviewing techniques included guidance on when to probe and what probing questions to focus on, as well as avoiding introducing bias into the interviews. In addition to practice during the classroom training, field practice was conducted after which the group reviewed each audio recording together. In each country, the team was also supervised by a field manager who managed the workflow and attended some interviews to reinforce training lessons and for quality assurance. The field manager made an introductory call or visit to each selected facility to explain the study, and interviewers obtained consent from each provider who participated at the time of the interview. Participating providers were given approximately 5 USD worth of phone credits to thank them for their time.

The semi-structured interview guide was developed during pre-testing in Kenya in April and June 2015, and modified for the Ghanaian context during training in Ghana in October 2015. We kept modifications to the Ghana guide to a minimum to maintain as much comparability as possible between the two countries. The interview guides covered three main topics: participation in the franchise network, participation in financing and quality improvement interventions offered by the AHME program, and a section on the facility’s experience with the relevant insurance scheme in the country. In the first two sections, respondents were asked about their facility’s reasons for (not) joining each intervention component, their interaction with the program staff conducting recruitment for the intervention, and their views of the benefits and disadvantages of each intervention component. Those whose facilities joined the intervention component were also asked about their experiences and what had changed in the facility since taking up the intervention. The section on NHIS/NHIF covered reasons for (not) applying for insurance accreditation, knowledge of the accreditation process and requirements, experience with the accreditation process for those who had ever applied, the benefits and challenges of being in the scheme, and perceptions of the insurance scheme, including the prevalence of insurance coverage among people in the facility catchment area. The full interview guides for Kenya and Ghana, respectively, can be seen in S1 and S2 Text. Most interviews lasted between 45 minutes and one hour.

### 2.3 Data analysis

Interviews were digitally recorded and transcribed by a local team in each country. The large majority of interviews were held in English; the few interviews held in Twi in Ghana or Kiswahili in Kenya were simultaneously translated and transcribed into English. A randomly selected section of each interview was back-checked to ensure transcription and, where needed, translation accuracy.

We used an inductive, thematic approach to coding and analyzing the interviews because, particularly in the case of Kenya, there was little existing literature on private providers’ experiences with the NHIS or NHIF from which to derive prior theories. An initial coding scheme was created by the first author based on thematic coding of a sub-set of the interviews from each country. Each interview was coded using an open coding approach, in which codes were derived from the data, and then common codes were identified across the interviews and grouped into code families and sub-codes. For instance, codes for “customer demand,” “financial reasons,” “government push” and “policy” were all grouped under the code family “Why apply (for) NHI” that captured facilities’ reasons for applying for NHIF or NHIS accreditation.

The authors and a research assistant then reviewed the initial codebook together to ensure common understanding of codes and consistency in how the codes were being applied. The analysis team then coded the interviews in Atlas.ti, continuing to use a thematic, open-coding approach in which additional codes were added when the existing codebook did not capture the content of the interview. For instance, to the code family “Why apply (for) NHI” additional sub-codes were added during coding for “competition from other facilities,” “gives clients confidence,” and “so clients can pay.” Each analyst coded a separate set of interviews; to increase reliability and validity during the coding process, the codebook was stored on a shared drive so that all team members could see updates as they were added, and bi-weekly team meetings were held in order to discuss new codes and merge codes that the team decided captured the same theme. For example, returning to the “Why apply (for) NHI” family, the subcodes for “policy” and “government push” were merged into one code reflecting that a facility applied for accreditation (in part) because of a government policy of encouraging facilities to enroll in the system. The final codebook with definitions can be seen in supplemental information file S5 Text. The first author also reviewed a sub-set of coded interviews to check for consistency across coders. The coding process indicated that saturation was reached on the health insurance scheme topics in both countries, as very few codes were added to the codebook during the later stages of the coding process.

### 2.4 Ethics

The study received ethical approval from the University of California San Francisco Committee on Human Subjects Research, the Kenya Medical Research Institute and the Ghana Health Service Ethical Review Committee. Informed consent was obtained from each respondent by the interviewer prior to beginning the interview. According to the requirements of the local ethical reviews, written consent was obtained from respondents in Ghana and verbal consent from respondents in Kenya (consent forms available in S3 and S4 Text). Verbal consent was approved in Kenya because the study was deemed to be low risk and respondents were not asked to provide identifying information.

## 3. Results

### 3.1 Characteristics of facilities and respondents

Of the 79 facilities included in the final sample, 52 were in Kenya and 27 in Ghana. In Kenya, facilities were distributed across Nairobi (N = 19), Rift Valley (N = 16) and Eastern (N = 17) regions, and in Ghana facilities were located in the Greater Accra (N = 11), Eastern (N = 10) and Western regions (N = 6). Multiple staff were interviewed at five facilities in Kenya and three facilities in Ghana because different staff members were better able to speak about different aspects of the facility’s operations. For example, the primary staff member who dealt with NHIS or NHIF was sometimes different from the staff member who made decisions about social franchising.

Facilities varied in terms of type, size, and the qualifications of the staff member(s) interviewed. In both countries the smallest facilities had only one full-time medical staff, whereas the largest facility in Ghana reported having 29 full-time medical staff and in Kenya 70 full-time medical staff. Although there was greater variation in the size of the facilities in Kenya, the median number of full-time medical staff (3) was smaller than in Ghana (8). The facility staff interviewed also had varying qualifications, although nurses and midwives predominated. In Kenya, respondents included five doctors, 24 nurses, 21 other medical staff (primarily clinical officers and auxiliary nurses) and seven administrators. In Ghana, respondents included five doctors, nine nurses, nine midwives, four other medical staff and two administrators. One respondent in Ghana did not answer the question about medical qualification.

As shown in Table 1, the majority of facilities in Kenya were clinics, with nine maternity homes and small numbers of health centers, hospitals and a dispensary included. In Ghana in contrast, nearly half of the facilities were maternity homes and the remainder were clinics or hospitals. Whereas 20 of the 27 facilities in Ghana were NHIS accredited, only 18 of the facilities in Kenya had NHIF outpatient accreditation. Of those 18 facilities, 14 also had inpatient accreditation; none of the facilities in the sample had inpatient accreditation only. The large majority of the facilities that had no NHIF accreditation were clinics, whereas higher-level facilities, particularly hospitals and maternity homes, were mostly accredited.

**Table 1:** Distribution of the study sample by country, facility type and health insurance accreditation status.

	Ghana		Kenya		
Facility type	Total sample	NHIS accredited	Total sample	NHIF inpatient accredited	NHIF outpatient accredited
**Hospital**	6	5	5	5	5
**Maternity home**	12	10	9	7	7
**Health center**	-	-	4	2	3
**Dispensary**	-	-	1	0	0
**Clinic**	9	5	33	0	3
**Total**	27	20	52	14	18

There were also country differences in respondents’ estimations of the percentage of their clientele that was enrolled in the relevant health insurance scheme (we did not check administrative records to verify this information). In Kenya, estimates varied widely from only 10 percent of clients to as much as 90 percent with NHIF coverage, although there was a sense among many respondents that coverage levels were increasing. In contrast, in Ghana respondents consistently said that about 80 percent of their clients had NHIS coverage, with a few estimates reaching as low as 60 percent or as high as 100 percent.

### 3.2 Major themes and overview of results

Major themes that emerged during the course of interviews with providers in both Kenya and Ghana included reasons for seeking or not seeking SHI accreditation, and the benefits and challenges of working with the SHI system (see full codebook in S5 Text). Other major themes differed somewhat in the two countries; in Kenya, the complexities of the NHIF accreditation process emerged as a major barrier to providers’ participation in the scheme, leading us to develop code families for the accreditation process, accreditation payment, reasons for having considered (but not applied for) accreditation and NHIF accreditation requirements. In Ghana, there were less complex code families only for accreditation process and requirements. On the other hand, there were major themes in Ghana related to lengthy reimbursement delays from the NHIS, including the effects of delayed payments on the facility, as well as a a new biometric system, which did not emerge from the Kenyan interviews. Other minor codes that were different across contexts included a code for the need for client awareness of NHIF in Kenya, and a code related to considering withdrawing from NHIS in Ghana.

In our discussion of the interview findings, we first discuss themes related to providers’ decisions about whether or not to apply for accreditation and their experiences with the accreditation process, as this was a specific objective of the study. We then turn to the major themes related to benefits and challenges of the system, comparing and contrasting between Ghana and Kenya to draw broader lessons about private providers’ participation in SHI systems.

### 3.3 The decision to apply for health insurance accreditation

**Fig 2:**
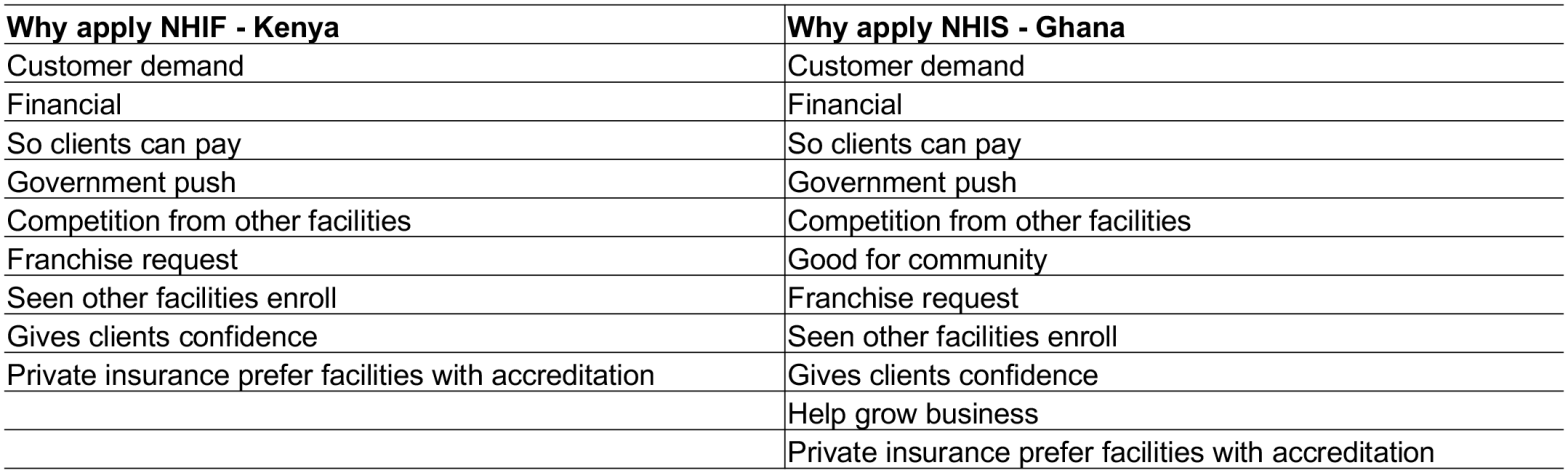
Providers’ reasons for applying for NHI accreditation.

As suggested by the breakdown of facilities by health insurance accreditation status in Table 1, respondents’ experiences with accreditation in Kenya were quite a bit more variable than in Ghana. See Fig 2 for a description of the major themes that emerged when providers described their reasons for applying for NHI accreditation.

The most common reason providers of all types in Kenya gave for applying for NHIF accreditation—whether or not they had completed the process at the time of interview—was because of increased demand from their clientele to accept the NHIF card. This was also commonly cited by unaccredited providers across facility types as a general reason for why a facility might want to apply for NHIF accreditation. Respondents noted that NHIF had become increasingly popular with the general public, in contrast to previous years when the scheme only covered civil servants.

*…most of our clients they were having [an] NHIF card, and when we failed to offer them [services], turning them away, it was not really very good. We would be losing so many clients, so many patients. So that’s why we decided to enrol for the NHIF*.

–Health center, Kenya, Rift Valley, franchised, NHIF outpatient accredited

In some areas, particularly in Nairobi, client demand for NHIF services was sufficiently high that providers felt that they had to apply for NHIF accreditation in order to remain competitive in their local market.

*…again there is competition all over. I mean people would choose to go to an NHIF accredited facility and not come here as much as possibly our quality is better than that place*.

–Health center, Kenya, Nairobi, franchised, NHIF inpatient and outpatient accredited

Respondents at some of the facilities that enrolled in NHIF due to the pressure of their local markets also cited financial motivations, noting that NHIF accreditation led to an overall increase in their client loads and thus greater profit for the facility. In several cases, respondents also said that NHIF enrollment would lead to greater profits for the facility due to the inability of clients in their catchment area to pay for services out of pocket. In this context, the spread of NHIF not only allowed clients to seek care more promptly, but reduced the need for facilities to provide services on a sliding scale or credit that was not always repaid.

*The people who cannot afford to pay cash and they are members of NHIF then we know we can claim some money from the NHIF. So there was also a business part of it there*.

–Maternity home, Kenya, Nairobi, franchised, NHIF inpatient and outpatient accredited

In the facilities in Ghana that were NHIS accredited, respondents from across the different types of facilities emphasized client-side reasons for joining the scheme even more strongly than in Kenya. More consistently than in Kenya, respondents in Ghana emphasized that if they did not accept NHIS, they would lose clients and their facilities would go out of business.

*I decided to join them because, in this area, when you don’t have the National Health Insurance accreditation, nobody will come to your facility*.

–Maternity home, Ghana, Eastern region, franchised, NHIS accredited

As in Kenya, some respondents in Ghana also linked the need for NHIS accreditation to the fact that their catchment areas were largely poor, and clients could not afford services without health insurance. In a few facilities that appeared to be in more underserved areas, respondents even said that NHIS specifically came to them to encourage them to join the system.

*I had to agree because of the clients. Because if I don’t have it [NHIS], look at this community, they would suffer…if I’m not having insurance they have to travel far away to another place*.

–Maternity home, Ghana, Western region, franchised, NHIS accredited

*It’s a very, very deprived area, you can see it…Our aim is not about money otherwise, I would have opted out [of NHIS] because most of our colleagues have opted out. But our aim is to assist the people here and that is why we continue to be in…we would have left long ago because the way they treat us*.

–Hospital, Ghana, Accra, franchised, NHIS accredited

This aspect of helping clients, along with the strong client pressure to accept NHIS coverage, led a number of respondents, such as the one quoted above, to explain their reasons for joining NHIS more in terms of the benefit to the community than to the facility. Although fewer mentioned profit motivations for joining NHIS as compared to providers in Kenya, a few respondents in Ghana did also say that payment under NHIS was at least guaranteed, if, as discussed below, very delayed.

Whereas respondents in facilities that were accredited or had applied for accreditation were quite consistent in citing client demand as their primary motivation for joining NHIF or NHIS, reasons for not joining the schemes were more varied. In Kenya, these reasons reflected the substantial variation in respondents’ estimates of the percentage of their clientele with NHIF coverage. Respondents in several lower-level facilities, primarily clinics, said that they had not applied for accreditation because their clients were not enrolled in NHIF, and participation therefore held no benefits for the facility.

*I have not applied…Most of my clients as I told you, only one in ten would could come and inquire about NHIF and nine of them they pay for the services that they receive*.

–Clinic, Kenya, Rift Valley, franchised, not NHIF accredited

Another group of respondents, particularly among those in Western Kenya, said that they had never considered NHIF accreditation because they did not know anything about the system or how it worked, or that they had not applied because they were not sure where to start with the process. Several also mentioned that their facility did not provide inpatient services, and seemed unaware of the newer outpatient scheme.

*Yes, I’ve thought about it, only that maybe … to find time and find somebody I can get proper information [from] then I would start off the issue… most of my colleagues around I’ve not seen any with it. I don’t know where to start, I just don’t know where to start*.

–Clinic, Kenya, Rift Valley, franchised, not NHIF accredited

I’ve not been interested because…when I put up an in-patient service, maybe I’ll get into it. But right now I’ve not been interested to know because I don’t have [inpatient services]

–Clinic, Kenya, Rift Valley, franchised, not NHIF accredited

Nearly all of the facilities that had not applied for accreditation either due to lack of information or not offering inpatient services were also clinics. This reflects both the fact that clinics constituted the largest group of facilities without accreditation, and that they are lower-level facilities that were least likely to be able to meet NHIF requirements prior to the introduction of the outpatient scheme.

In Ghana, there were few facilities without NHIS accreditation, and several of those were in the process of entering the scheme or wanted to make improvements to the facility before going through the accreditation inspection process. Only one respondent provided a reason for actively choosing not to join NHIS, which was that the facility wanted to maintain a middle class clientele and avoid overcrowding.

### 3.4 Experiences with the accreditation process

The process for gaining NHIS accreditation in Ghana was described as straightforward by most respondents. There were some complaints about the length of the accreditation process, with a few respondents reporting that it took their facility up to a year. Several respondents also noted that it was burdensome to obtain the papers or copies of other licenses and certificates required to apply for accreditation. Yet overall, challenges that facilities had experienced with the accreditation process appeared to be related to individual circumstances, for example needing to obtain certain items of equipment, a water connection, the permits for the building, or additional staff, rather than broader systemic issues or the type of facility.

**Fig 3:**
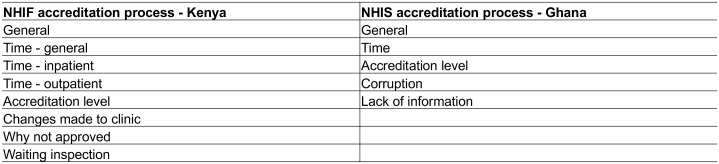
Provider descriptions of the NHI accreditation process.

In Kenya, in contrast, the accreditation process emerged as a major barrier to private healthcare providers’ participation in the scheme. Fig 3 summarizes the codes related to this topic. Respondents described a lengthy process for accreditation, in which the NHIF requirements were not always clear or were difficult to meet, and in which their case was delayed at multiple points in the process. Facilities frequently reported struggling to meet the accreditation requirements in terms of equipment and having enough space or rooms for the required services. This was particularly true for facilities that rented their buildings and could not undertake new construction.

*Mostly the structure because this structure is rented, it’s just a small room, and when I want to integrate [into] the NHIF I require a bigger room, yes a big space so that one will be the most challenge*.

–Clinic, Kenya, Eastern region, franchised, not NHIF accredited

Respondents also mentioned extensive lists of areas that NHIF checked during the accreditation inspection, ranging from infrastructure, to staff qualifications and human resources record keeping, to specific instruments or cleaning items, to client information and comment cards. Facilities of all types, including the higher-level hospitals and maternity homes, noted that the accreditation requirements were challenging and they had to make changes in response to the NHIF inspection.

The extensiveness of the checklist and inspection process posed two challenges for the facilities. First, meeting the requirements required at times substantial financial investments with an uncertain timeline for a return in the form of accreditation.

*Yes, every department they do rate, if you they’ll tell you “you are failing in the side of transport,” “you are failing in the side of maybe record keeping,” “you are failing in the side of maybe kitchen”, “you are failing in the side of maybe drainage.” …You need to do this and this they are going to give, maybe they are going to give the time framework when you need to improve*.

–Maternity home, Kenya, Nairobi, franchised, NHIF inpatient and outpatient accredited

*It all boils down to finance availability, if they [NHIF] want you to have this and you don’t have the money to have it, it’s all useless*.

–Maternity home, Kenya, Nairobi, franchised, NHIF inpatient and outpatient accredited

Second, the inspection process was a point at which some facilities stalled in the accreditation process, either waiting for an inspection, because they did not continue the process after failing the first inspection, or because they did not receive feedback on the inspection to begin with.

*I applied in 2012, I didn’t get any response. So I stopped the process until recently, last month they called me. They told me to take the paper and re-apply…In 2012, they came, did an assessment we never got feedback. Nobody came back to us, it just went quiet and that was it*.

–Clinic, Kenya, Eastern region, franchised, NHIF outpatient accredited

In a few cases, respondents also reported NHIF officers directly asking them for bribes in order to complete or speed up the accreditation process.

We have [considered applying], in fact we have tried twice but they have never considered us… Because in the year 2013, the NHIF personnel were going round and what they needed was Ksh250,000 to register you. We were wondering where that money was going but they said it was “for their pockets” if you want them to consider you…

–Clinic, Kenya, Rift Valley, franchised, not NHIF accredited

These administrative challenges with the accreditation process were again reported by facilities of different types. Such stories of NHIF officers requesting kickbacks, or the implication that delays in the accreditation process were sometimes intentional, may have contributed to some providers’ suspicion about NHIF and the general idea of joining the scheme. Some providers—in this case primarily the lower-level clinics—even cited the difficulty of meeting NHIF requirements and the cumbersome accreditation process as reasons for not applying for accreditation in the first place.

*I’ve never considered because I see it as if it’s a bit difficult. I don’t know, anything to do with the government…I can recall how I suffered when I was trying to register the clinic. So we are going also to apply also for NHIF, you struggle so much and later see no much benefit*.

–Clinic, Kenya, Rift Valley, not franchised, not NHIF accredited

*…two years ago we wanted [to apply]…but we saw it’s a bit cumbersome so we left it…because when you don’t know the way in, you may not know the way out, so we just left it*.

–Clinic, Kenya, Rift Valley, franchised, not NHIF accredited

On the other hand, several respondents who had successfully joined NHIF did credit the extensiveness of the accreditation process with having led to improvements in their facilities in terms of quality and scope of services, and upgrades to equipment and infrastructure.

### 3.5 Benefits and challenges of participation in the health insurance scheme

**Fig 4:**
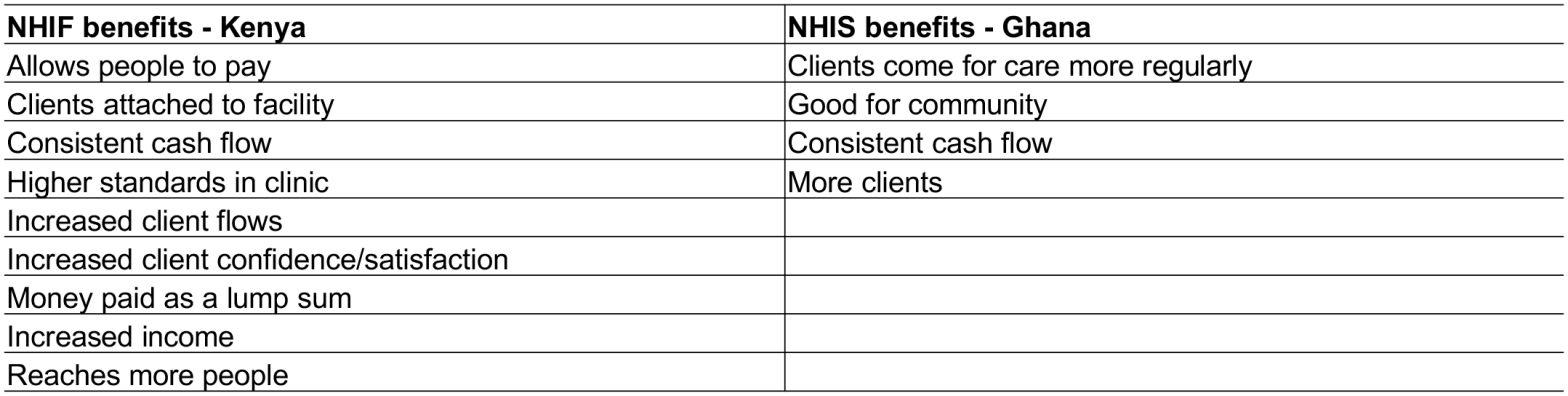
Perceived benefits of NHI accreditation.

Respondents in both countries widely agreed that the respective health insurance scheme is good for the country, and particularly for healthcare seekers (see Fig 4 for code summary).

*… you see the good thing with NHIF is that it is starting from down there upward… I cannot say that this one I will not give him service because he or she is poor, I will only give this one because he or she is able to pay. So you see now so that’s why NHIF can succeed*.

–Dispensary, Kenya, Rift Valley, franchised, not NHIF accredited

**Fig 5:**
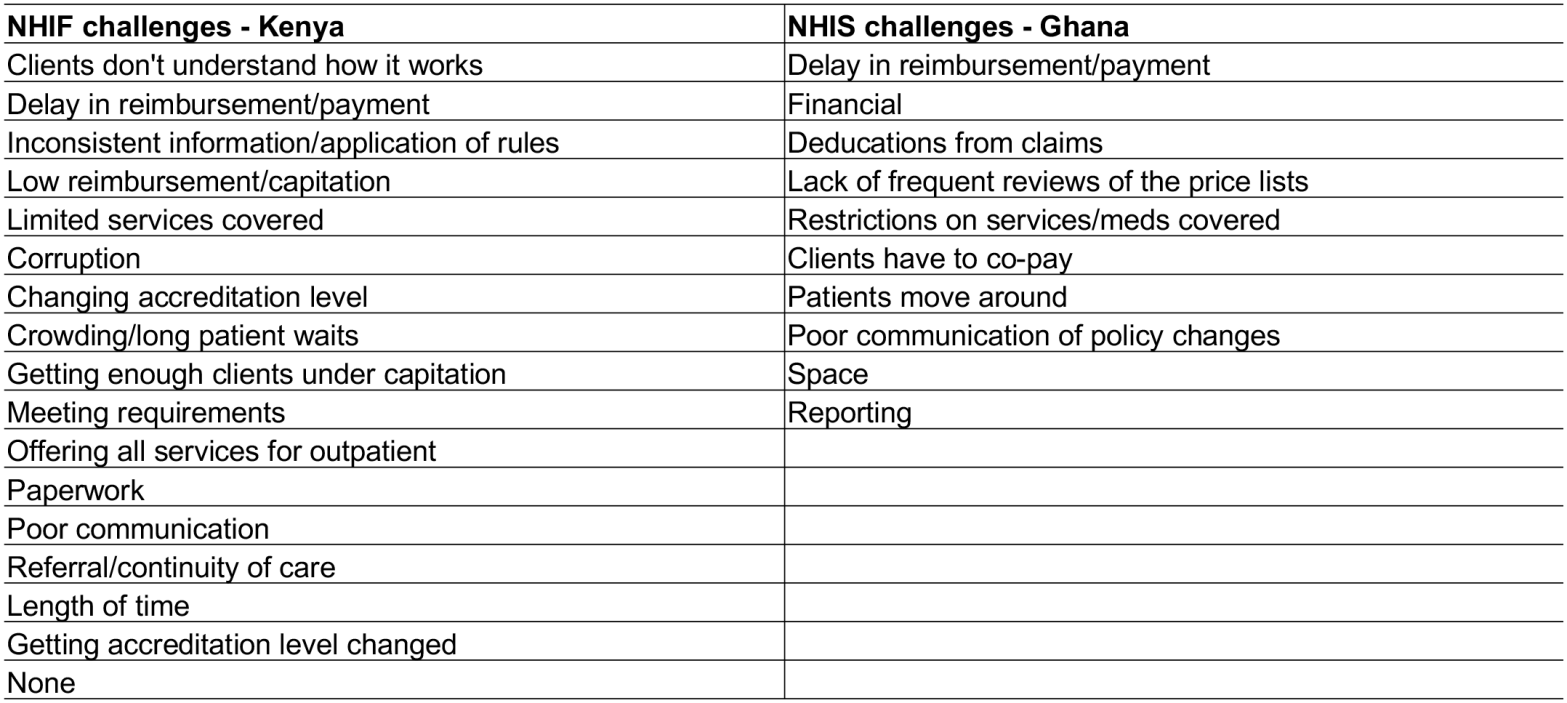
Perceived challenges of NHI accreditation.

At the same time, providers from all facility types in Ghana were very dissatisfied with the functioning of the NHIS due to the issue of delayed claims reimbursement (see Fig 5 for code summary). At the time of data collection, most providers reported a delay of six to eight months in being paid for their claims.

*We are in October*, *fortunately*, *they have paid us up to April*. *So they’ve done very well… Next two weeks we will be in November*. *So you can imagine how long it takes to pay us*. *[It] depends on them we cannot predict [whey they will pay]*.

–Hospital, Ghana, Accra, franchised, NHIS accredited

Providers cited a number of challenges in their clinic operations that resulted from the long delay in receiving claims payments from NHIS. The most common of these was difficulty in stocking medicines; facilities needed to have medicines in stock for regular dispensing from the pharmacy, but with the delay in reimbursements could not always afford to restock as needed. Many providers also said that they purchased medicines on credit from pharmacies or pharmaceutical suppliers. Other commonly reported challenges included difficulties in paying employees and other regular bills.

*You get a lot of clients a day [but] when it comes to the other side of it, money doesn’t come as [fast as] we use the drugs. So it makes business very hard because at times we credit the drugs from our suppliers, and the drugs finish but there is no money to pay for [more] drugs, and when you order for some they would tell you [you] are owing*.

–Hospital, Ghana, Accra, franchised, NHIS accredited

In order to cover the regular operating costs while waiting for NHIS reimbursements, providers relied on several other sources of funds, including cash payments from the few clients who were not covered by NHIS, using their personal money, and taking loans. The unpredictability of reimbursements was such that several respondents said that the delay in NHIS claims payment was a significant source of stress in their businesses. A few providers, such as the one quoted above, even said that they would have withdrawn from NHIS due to delayed payments if it were not for the pressure from their clients to accept the insurance.

Other challenges that providers reported with regards to NHIS were minor in comparison, and included deductions from reimbursement claims for services not covered, and that tariff rates for medicines were below their market price. Some providers in lower-level facilities, such as maternity homes, also felt that NHIS eligibility restrictions on what services they could be reimbursed for under the scheme were unfair.

*There are some of the medicines which especially we the midwives are not allowed to give…Recently I was at a meeting when we were trying to revise the standard treatment guidelines…we were telling them [NHIS] to try as much as possible to think of the midwife who is in the remotest area trying to help, so then you give the medicines and they [NHIS] said, “we will not pay” [reimburse]*.

–Maternity home, Ghana, Eastern region, franchised, NHIS accredited

As indicated by the quote, these providers felt that they had to offer a broader set of services and medicines than those covered by NHIS due to the lack of other options for their clients.

Kenyan providers who were accredited by NHIF reported a more varied set of challenges with participation in the scheme. Some Kenyan providers similarly complained that NHIF takes too long to reimburse providers, although the time to reimbursement appeared to be much shorter than in Ghana, at around two to three months. A number of providers were also dissatisfied with the rates that they were paid by NHIF. In particular, providers across facility types that had outpatient accreditation complained about low capitation rates, saying that this affected their ability to provide services.

*…outpatient [is] by capitation method… [NHIF] expect you to be given Kshs.712 for three months [for a client] to be treated in a private hospital. It can’t work. And there they indicate you [the client] can be treated as much as you can within those three months*.

–Hospital, Kenya, Rift Valley, franchised, NHIF inpatient and outpatient accredited

In relation to reimbursement rates and other key aspects of the scheme, respondents in Kenya also indicated that they did not think the NHIF listened to their concerns or involved them adequately in decisions. This concern was voiced by providers across facility types, but primarily among those in Nairobi whose facilities had been participating in NHIF for a longer period of time. They said that NHIF did not have clear communication channels with providers, such that they did not know to whom in the NHIF offices to address questions or complaints. Poor communication was also a concern for providers in relation to information that they thought NHIF should cascade down to them, such as new policies or changes in rates.

NHIF would be a very, very nice program if they stick to their word and also if they listen to facilities because we are the people on the ground. We are the ones who are offering this service… they should listen to us and what we tell them as little facilities. When we tell them that this amount is very little, let them sit down with us and let’s reach an agreement…

- Maternity home, Kenya, Nairobi, franchised, NHIF inpatient and outpatient accredited

Providers also said that NHIF should do more to sensitize clients to how the health insurance system works. They said that clients often did not know which services NHIF covered, and did not understand requirements for coverage of their dependents or how the primary provider system worked under the outpatient capitation scheme.

*There is a disconnect in what clients believe NHIF does and what we offer because the client thinks when they are under NHIF, they usually think they are entitled to everything…so when they come and we tell them ‘we don’t do that here you have to go elsewhere’ it may bring issues*.

–Clinic, Kenya, Rift Valley, not franchised, not NHIF accredited

This was similarly a concern across provider types. Providers said that clients thus had inaccurate expectations regarding the services that would be covered under their NHIF enrollment, and providers—in their position as clients’ first-line points of contact within the NHIF system—would face these frustrations.

## 4. Discussion

The expansion of social health insurance schemes can be an important means toward achieving universal health coverage, particularly if strategies to expand SHI are targeted towards poor populations [1,10]. Since the private sector is an important source of healthcare for the poor in many LMICs, including in Sub-Saharan Africa [14], ensuring that the private sector is effectively incorporated into SHI systems is key to achieving this goal [15]. Our research with private healthcare providers in Ghana, which has one of the better-established social health insurance systems in Sub-Saharan Africa, and Kenya, which is seeking to expand the NHIF into a broad social health insurance scheme, suggests several common factors that should be considered regarding private providers’ incentives and motivations to participate in SHI schemes.

First, our findings demonstrate that private sector providers recognize the public health value of social health insurance and can be motivated to be active partners in SHI schemes. The most fundamental measure for leveraging the private sector to expand UHC through social health insurance is thus to ensure that SHI schemes are designed to include mechanisms for the broadest possible accreditation and participation of private providers. For instance, prior to the introduction of the NHIF outpatient scheme in Kenya, many private providers could not engage with the NHIF due to their scope of practice.

In addition, SHI schemes need to consider the incentives of for-profit private providers to gain accreditation and remain engaged in the health insurance scheme. Our findings from both Ghana and Kenya indicated that market pressure—i.e. client demand for accredited facilities that accept health insurance—was critical to incentivizing providers to join the health insurance scheme, as well as to remain in the scheme when operational difficulties were experienced. The importance of client demand is highlighted by the continued participation in NHIS of most of the interviewed providers in Ghana, despite the difficulties they experienced due to delayed reimbursements. In Kenya, in contrast, many providers had not even considered joining NHIF because they did not perceive this to be important to their clientele. This aspect of private providers’ motivations to join SHI systems has not been discussed in previous literature, and has important policy and programmatic implications.

In expanding systems such as the NHIF, client enrollment drives should thus be accompanied by efforts to demonstrate to private providers, and particularly those in lower-level facilities, how they may benefit from this shift in their market. Coordinated approaches to increasing enrollment among hard-to-reach populations, such as informal sector workers, alongside the private facilities that serve these populations, are needed in order to ensure that service availability—in the form of more accredited facilities—increases along with client enrollments. While literature has demonstrated that patient awareness campaigns and support are important to increase overall SHI uptake [43,44], our findings suggest that greater education and awareness among both providers and patients is important to ensuring the successful growth of emerging health insurance markets. Although the existing literature on private provider experiences with SHIs is very limited, these findings are in line with a study in Karnataka, India, which found that lack of coordinated rollout and alignment of incentives among providers (including private providers) and patients negatively impacted social health insurance implementation in the state [45]. In a context such as Kenya, where the NHIF was still poorly understood by many providers and (according to providers) their clients, basic awareness raising among both providers and the general population is therefore a key first step, particularly as capitation is introduced.

In Ghana, there is also the critical issue of how delayed reimbursements impact private providers’ business models. Consistent with other studies, providers in our sample reported facing long delays in receiving payments from NHIS, which affected their ability to run the facility [16,33,35]. This problem may hit private providers especially hard since, unlike public sector facilities, they are not able to rely on government payment of salaries. Other studies in Ghana have suggested that the problem of delayed reimbursements has contributed to reported practices of providers charging insured clients informal fees or providing them with lower quality care than insured patients. However, these studies were based on interviews with clients and NHIS staff as well as providers, and it was unclear if providers themselves admitted to this practice [16,33]. We did not find evidence in this study that providers charged NHIS clients informal fees, but this may be due to the fact that our study included providers only, who are likely reluctant to report a practice that goes against NHIS policy. Private providers did indicate that they relied on payments from uninsured clients to bridge the financing gap caused by delayed reimbursements; such uninsured clients may be less likely to attend public facilities. In addition to the impact on their businesses, delayed reimbursements were a major source of dissatisfaction with the NHIS among providers, as has been found in studies incorporating both public and private providers [35].

Apart from their business models, our findings suggest that the administrative burden of health insurance procedures may be a disincentive for private providers to participate in the scheme. Administrative challenges that providers face may be compounded by unclear lines of communication with SHI offices; some providers in Kenya felt that the NHIF did not give them reliable information or provide clear communication channels for answering questions or addressing complaints, particularly during the accreditation process. Among unaccredited providers, lack of information and a general sense that working with the NHIF can be difficult dissuaded some from applying for accreditation in the first place, which is a key barrier to expansion of the system. In October 2016, NHIF announced that it was eliminating accreditation fees for private facilities seeking to join NHIF and has simplified the assessment procedure [46]. This is an important step towards addressing some of private providers’ concerns with the accreditation process, but further monitoring and research will be needed in order to assess the degree to which these measures facilitate accreditation and increase transparency.

Providers’ discussions suggested that engaging them more actively as stakeholders in the SHI scheme, and providing forums where their concerns can be heard and addressed, may help to address dissatisfaction over issues such as delayed reimbursement and accreditation processes. Perceived lack of voice in the health insurance scheme was a complaint among providers in both Ghana and Kenya, and contributed to the sense among some that they were not benefiting much from the scheme. In a recent study, Alhassan et al. [35], for instance, similarly found low levels of satisfaction with information provision and feedback channels from NHIS in Ghana. Yet they found that systematic community engagement programs in Ghana helped to improve provider perceptions of NHIS by promoting providers’ interests and encouraging their active participation in the system. These interventions involved eliciting feedback from community members on service quality among both health service providers and the NHIS, and then engaging providers, insurers, and district-level policy makers in follow-up discussions with community representatives. This is a lesson that could be carried over to Kenya, where mistrust of the NHIF still appeared to be quite high among many private providers.

At the same time that this study highlights important common considerations for incorporating private sector providers into SHI systems across Sub-Saharan Africa, there were differences in providers’ experiences in Kenya and Ghana that are likely related to the structure and maturity of the national SHI system. While the NHIF in Kenya has only rolled out to the population beyond civil servants somewhat recently, Ghana’s NHIS has been active for well over a decade. Having lived longer with a (theoretically) compulsory scheme, patients in Ghana are therefore more likely to be enrolled in the SHI than their counterparts in Kenya, explaining the stronger market pressure for provider accreditation in Ghana. Further, the perceived complexity of the accreditation process among Kenyan providers may have been due, in part, to lack of familiarity with the NHIF and the fact that the opening of the system to facilities providing outpatient services only was fairly recent at the time of the data collection.

There are several limitations to this study that should be kept in mind when interpreting the results. First, our findings are based on a sample drawn primarily from facilities that were members of social franchises. Eligibility criteria for participation in a social franchise often require that facilities have a baseline level of quality, and franchise membership entails trainings and quality improvement measures. Therefore, these franchised facilities are likely to have higher quality than other private sector facilities, which may make it easier for them to gain health insurance accreditation. We were also unable to triangulate providers’ direct reports of their experiences with the health insurance scheme with facility or NHIS/NHIF administrative records regarding client volumes, facility finances, or the accreditation process. We also did not interview the facilities’ clients—whether insured or uninsured—and therefore cannot address differential treatment of health insurance clients, which has been noted to be an important issue in Ghana in particular [16,33,34].

## 5. Conclusions

Engaging private providers is critical to achieving UHC in many countries, and better understanding of how private providers interact with social health insurance schemes is an important step towards designing policies for this aim. To the best of our knowledge, this study is the first to focus on the experiences of private sector providers with social health insurance systems in Sub-Saharan Africa. It is also the first study to examine providers’ experiences with NHIF in Kenya, which is important as the system seeks to expand both population and services coverage. Our findings demonstrate that private providers recognize the benefits of SHI to their facilities and the population at large. However, they face challenges and disincentives to engaging with SHI schemes that, if not addressed, may lead to reduced trust in the health insurance system and lower motivation to participate. Factors related to the structure of SHI schemes, their level of development and both client and provider understanding of the system contribute to the differences in the challenges providers face in engaging with their national system. It is therefore important that further country-specific research be carried out in LMICs that are establishing or expanding SHI systems in order to work towards more effective involvement of the private sector in social health insurance.

## Supporting information

S1 Text Kenya interview guide. Kenya provider interview guide.
(DOCX)

S2 Text Ghana interview guide. Ghana provider interview guide.
(DOCX)

S3 Text Kenya verbal consent form. Kenya verbal consent form.
(DOCX)

S4 Text Ghana written consent form. Ghana written consent form.
(DOCX)

S5 Text Codebook. Codebook.
(DOCX)
