# Supplementary material for: Private healthcare provider experiences with social health insurance schemes: Findings from a qualitative study in Ghana and Kenya
Source: PLoS One. 2018 Feb 22;13(2):e0192973. doi: 10.1371/journal.pone.0192973 (PMC5823407; doi:10.1371/journal.pone.0192973)
Supplement: S1 Text — Kenya provider interview guide. (DOCX) [file pone.0192973.s001.docx]

**In-Depth Interview Field Guide: AHME Providers Round 2**

_________________________________________________________________________________________________

**Session ID:** [ ] [ ] [ ] **Date (DD/MM/YY)**: ____ /____ / ____

Name of Interviewer: _____________________________________________________________

Name of Clinic: _____________________________________________________________________

Village/City and County: ___________________________________________________________

**Intervention/NHIF participation**

____________________________________________________________________________________________

Check the facility selection list and confirm all information with the provider. Note any discrepancies in the margin and explain reason.

Franchise Network 🞏 AMUA 🞏 TUNZA

When did the facility join the network? Month __________ Year ______________

MCF 🞏 YES 🞏 NO

SafeCare 🞏 YES 🞏 NO

NHIF Inpatient 🞏 YES 🞏 NO

NHIF Outpatient 🞏 YES 🞏 NO

**Introduction and consent**

____________________________________________________________________________________________

1. ***Introduce yourself and the study***
2. ***Obtain written informed consent [READ WRITTEN CONSENT FORM]***
3. Did you answer any questions? 🞏 YES 🞏 NO
4. Did participant agree to participate? 🞏 YES

🞏 NO 🡪 *STOP INTERVIEW*

1. Did you date and sign consent? 🞏 YES 🞏 NO
2. Did you give participant a copy of consent? 🞏 YES 🞏 NO
3. ***Obtain permission to tape record***

We would like to tape-record the interview so that we can remember everything we discuss here today.

1. Is it okay with you if I tape-record? 🞏 YES 🡪 *TURN ON RECORDER*

🞏 NO 🡪 *TAKE NOTES*

1. ***Turn on tape recorder and say session ID***

**I. Social franchising**

1. How did you first learn about Amua/Tunza?
2. What did the Amua/Tunza recruiter say to you when they came to explain the program? *Probe*: What questions did they ask you? What did they check about the facility?
3. At the time the recruiter came, what did you see as the benefits of joining Amua/Tunza? What did you see as the potential challenges of joining?
4. What has changed in the clinic since you joined Amua/Tunza? *Probes:* Equipment, commodity supply, record keeping, branding, services added, others
5. Did you provide services for child health before joining Amua/Tunza?
   1. Which services did you provide before joining Amua/Tunza?
   2. Have you added any new child health services since joining Amua/Tunza? Why did you add those services?
   3. Have you changed the way you provide child health services? How?
   4. Did you participate in Amua/Tunza training on IMCI?
      1. If yes: What did you learn in this training?
   5. Can you give me an example of a recent client that you treated differently than you would have before the IMCI training?
6. Did you provide reproductive health services before joining Amua/Tunza?
   1. Which services did you provide before joining Amua/Tunza?
   2. Have you added any new reproductive health services since joining Amua/Tunza? Why did you add those services?
   3. Have you changed the way you provide reproductive health services since joining Amua/Tunza? How?
   4. Did you participate in Amua/Tunza training on reproductive health?
      1. If yes: What did you learn in this training?
   5. Can you give me an example of a recent client that you treated differently than you would have before the reproductive health training?
7. Is there anything else you would like to tell me about your experience with Amua/Tunza so far?

*Check the cover sheet: Is the clinic participating in MCF and/or SafeCare?*

🞏 YES 🡺 Go to Section III

🞏 NO 🡺 Go to Section II

**II. Non-participation in MCF/SafeCare**

1. Have you heard of the Medical Credit Fund (MCF)?
   1. If no: Have you heard of PharmAccess and their loan program?
      1. If still no, go to question 9.
   2. How did you first learn about MCF?
   3. Did anyone come to explain the MCF program to you? Can you describe what they told you?
      1. *Probes*: What questions did they ask you?
      2. What did you see as the benefits of MCF? The potential challenges?
      3. Why did you not take a loan with MCF in the end?
   4. Have you ever taken a loan before?
      1. If yes: Where did you get the loan from? What did you use it for?
   5. How do you think the MCF program compares to your loan options from a regular bank? Why?
2. Have you heard of SafeCare?
   1. If no: Have you heard of PharmAccess and their quality improvement program?
      1. If still no, go to Section 4.
   2. How did you first learn about SafeCare?
   3. Did anyone come to explain the SafeCare program to you? Can you describe what they told you?
      1. *Probes*: What questions did they ask you?
      2. What did you see as the benefits of SafeCare? The potential challenges?
      3. Why did you not join SafeCare in the end?
3. What is the relationship between MCF, SafeCare and Amua/Tunza?

🡺 Go to Section IV

**III. Participation in MCF/SafeCare**

1. Now I would like to talk about the MCF program. What did the MCF recruiter say to you when they came to explain the program?
   1. *Probes*: What questions did they ask you? What did they check about the facility?
   2. What did you see as the benefits of taking a loan with MCF?
   3. What did you see as the challenges?
   4. Did you take a loan with MCF?
2. If did not take a loan: Why didn’t you take a loan?
   1. *Probes*: facility circumstances, program conditions
3. If took a loan with MCF: Why did you decide to take a loan?
   1. What was the process for obtaining the loan?
   2. What are you using the loan for? Why did you want a loan for this?
   3. Do you expect to take another loan after you finish repaying the current one? If so, what for? If not, why not?
   4. What are the benefits of participating in MCF?
   5. What are the challenges?
4. How do you think the MCF program compares to your loan options from a regular bank? Why?
5. What did the SafeCare recruiter say to you when they came to explain the program?
6. *Probes*: What questions did they ask you? What did they check about the facility?
7. At the time the recruiter came, what did you see as the benefits of participating in SafeCare?
8. What did you see as the potential challenges?
9. Did you join SafeCare?
10. If did not participate: Why didn’t you participate in SafeCare?
    1. *Probes*: facility’s circumstances, program conditions
11. If joined SafeCare: Why did you decide to participate in SafeCare?
    1. Could you describe the SafeCare assessment process to me?
    2. What are the gaps that SafeCare identified in order for your facility to improve?
    3. What support is SafeCare giving you to make those improvements?
    4. What else would help you to make those improvements? Why?
    5. What are the benefits of participating in SafeCare?
    6. What are the challenges of participating in SafeCare?
12. Is there anything else you would like to tell me about your experience with MCF or SafeCare so far?
13. What is the relationship between MCF, SafeCare and Amua/Tunza?

**IV. NHIF**

1. Now I would like to talk about NHIF. Are many people in this area enrolled with NHIF?
   1. Are many of your clients are enrolled with NHIF? (*Probe:* how many out of 10 have NHIF?)
2. For facilities that are **not** NHIF accredited: What do you know about the requirements for a facility to become accredited? *Probe:* inpatient vs. outpatient
   1. Which of these requirements would be most difficult for your facility to meet? Why?
   2. Has the facility ever considered applying for NHIF accreditation?
      1. If they have never considered it: Why not?
      2. If they have considered it but not applied: Why have you not actually applied?
         1. What kind of information/assistance would you need to apply?
         2. How could Amua/Tunza help you in applying for accreditation? What about MCF or SafeCare?
3. If facility **is accredited OR ever applied** for NHIF accreditation:
   1. Why did you decide to apply for accreditation? *Probe*: inpatient vs. outpatient
   2. Can you describe the process to become accredited?
      1. *Probes*: What were the steps? What were the requirements? How long did the accreditation process take?
      2. What were the challenges you faced during this process?
   3. If application was rejected/still pending: Why was your application not approved or why hasn’t it been approved yet?
   4. What kind of assistance would have helped you gain accreditation more easily?
      1. How could Amua/Tunza help you in applying for accreditation? What about MCF or SafeCare?
4. If facility is accredited: Now that the facility is accredited, how to you find participation in NHIF?
   1. What are the benefits of participating?
   2. What are the challenges?
   3. If you have any challenges with NHIF, how do you resolve them?
   4. Are the benefits and challenges different for inpatient and outpatient? How?
   5. How do you maintain your accreditation?
   6. How do your clients perceive NHIF? Why?
   7. How has joining NHIF affected your facility finances?
5. Is there anything else you would like to tell me about your experience with NHIF?

**Demographic Sheet: AHME Provider Round 2**

**Session ID:** [ ] [ ] [ ] **Date (DD/MM/YY)**: ____ /____ / ____

| No. | Question | Coding Categories |
| --- | --- | --- |
| 1 | Gender | 🞏 1. Male |
|  |  | 🞏 2. Female |
| 2 | How old are you (in years)? | \|___\|___\| |
| 3 | What is the highest level of education you have completed? | 🞏 1. Never went to school or less than completing primary |
|  |  | 🞏 2. Primary education |
|  |  | 🞏 3. Secondary education |
|  |  | 🞏 4. Vocational/trade school |
|  |  | 🞏 5. University |
|  |  | 🞏 6. Masters/doctorate |
| 4 | What is your technical qualification? | 🞏 1. Medical doctor |
|  |  | 🞏 2. Medical assistant |
|  |  | 🞏 3. Nurse |
|  |  | 🞏 4. Community health/auxiliary nurse |
|  |  | 🞏 5. Midwife |
|  |  | 🞏 6. Health assistant |
|  |  | 🞏 7. Clinical Officer |
|  |  | 🞏 9. Other 🡪 please specify ______________________________ |
| 5 | Are you the owner of this facility? | 🞏 0. No |
|  |  | 🞏 1. Yes |
| 6 | What is your position title? | ______________________________ |
| 7 | How long have you been practicing (in years)?  *(N/A if admin with no degree)* | \|___\|___\| |
| 8 | How long have you been working at this clinic (in years)? | \|___\|___\| |
| 9 | What type of facility is this clinic? | 🞏 1. Hospital |
|  |  | 🞏 2. Health center |
|  |  | 🞏 3. Clinic |
|  |  | 🞏 4. Maternity home |
|  |  | 🞏 5. Dispensary |
| 10 | What year did you join NHIF?  *(N/A if not accredited)* | __________________________________ |
| 11 | How many medical staff does this clinic have? (doctors, nurses, midwives, medical assistants/aides) | Full Time Locum  \|___\|___\| \|___\|___\| |
| 12 | How many non-medical (support) staff does this clinic have? (cleaners, security) | \|___\|___\| |
| 13 | What health services are offered at this clinic? *(check all that are applicable)* | 🞏 1. Family planning |
|  |  | 🞏 2. Antenatal/ANC |
|  |  | 🞏 3. Delivery |
|  |  | 🞏 4. Postnatal care/PNC |
|  |  | 🞏 5. Post abortion care |
|  |  | 🞏 6. STI |
|  |  | 🞏 7. HIV counseling, testing & management |
|  |  | 🞏 8. Malaria |
|  |  | 🞏 9. TB |
|  |  | 🞏 10. Diarrhea |
|  |  | 🞏 11. Respiratory tract infections |
|  |  | 🞏 12. Nutrition |
|  |  | 🞏 13. Immunizations |
|  |  | 🞏 13. Cervical cancer screening |
|  |  | 🞏 14. Other 🡪 please specify ______________________________ |
