# Supplementary material for: Private healthcare provider experiences with social health insurance schemes: Findings from a qualitative study in Ghana and Kenya
Source: PLoS One. 2018 Feb 22;13(2):e0192973. doi: 10.1371/journal.pone.0192973 (PMC5823407; doi:10.1371/journal.pone.0192973)
Supplement: S2 Text — Ghana provider interview guide. (DOCX) [file pone.0192973.s002.docx]

**In-Depth Interview Field Guide: AHME Providers Round 2**

_________________________________________________________________________________________________

**Session ID:** [ ] [ ] [ ] **Date (DD/MM/YY)**: ____ /____ / ____

Name of Interviewer: _____________________________________________________________

Name of Clinic: _____________________________________________________________________

Village/City and Region: ___________________________________________________________

**Intervention/NHIS participation**

____________________________________________________________________________________________

Check the facility selection list and confirm all information with the provider. Note any discrepancies in the margin and explain reason.

When did the facility join BlueStar? Year ______________

Has the facility:

Taken a loan from the Medical Credit Fund? 🞏 YES 🞏 NO

Had a SafeCare assessment? 🞏 YES 🞏 NO

Is the facility NHIS accredited? 🞏 YES 🞏 NO

**Introduction and consent**

_______________________________________________________________________________________

1. ***Introduce yourself and the study***
2. ***Obtain written informed consent [READ WRITTEN CONSENT FORM]***
3. Did you answer any questions? 🞏 YES 🞏 NO
4. Did participant agree to participate? 🞏 YES

🞏 NO 🡪 *STOP INTERVIEW*

1. Did participant date and sign consent? 🞏 YES 🞏 NO
2. Did you give participant a copy of consent? 🞏 YES 🞏 NO
3. ***Obtain permission to tape record***

We would like to tape-record the interview so that we can remember everything we discuss here today.

1. Is it okay with you if I tape-record? 🞏 YES 🡪 *TURN ON RECORDER*

🞏 NO 🡪 *TAKE NOTES*

1. ***Turn on tape recorder and say session ID***

**I. Social franchising**

1. How did you first learn about BlueStar?
2. What did the BlueStar recruiter say to you when they came to explain the program?
   1. What questions did they ask you?
   2. What did they check about the facility? *Probes:* look of the facility, size, records, license, others
3. At the time the recruiter came, what did you see as the benefits of joining BlueStar?
   1. What did you see as the potential challenges of joining?
4. What has changed in the clinic since you joined BlueStar?
   1. Have you added any new services since joining BlueStar?
   2. Are there any other changes to the clinic? *Probes:* Equipment, commodity supply, record keeping, branding
5. Did you attend the BlueStar training on IMCI?
   1. If yes: How have you changed the way you provide child health services since taking this training?
   2. Can you give me an example of a recent client that you treated differently than you would have before the IMCI training?
6. Is there anything else you would like to tell me about your experience with BlueStar so far?
7. Is there anything that BlueStar could improve on?
   1. Why is that important to your clinic?

**II. SafeCare/Medical Credit Fund**

1. Now I’d like to talk about the business side of running this facility. Have you participated in any business trainings since joining BlueStar?
   1. Which organization sponsored the training?
   2. How many days was the training?
   3. What did you learn in the training?
   4. Could you give me an example of how you used this information in the clinic?
2. Has anyone from the SafeCare quality improvement program or PharmAccess spoken to you about their program, or conducting an assessment of your clinic?
   1. If no: go to question 16
3. What did the SafeCare/PharmAccess representative say to you when they came to explain the program?
4. What do you see as the benefits of participating in SafeCare?
5. What do you see as the challenges of participating in SafeCare?
6. Did you agree to participate in SafeCare?
7. If did not agree to participate: Why didn’t you agree to participate in SafeCare?
8. If agreed to participate in SafeCare: Why did you decide to participate in SafeCare?
   1. Could you describe the SafeCare assessment process to me?
   2. What are the gaps that SafeCare identified in order for your facility to improve?
   3. What support is SafeCare giving you to make those improvements?
   4. What else would help you to make those improvements? Why?
9. Has anyone from the Medical Credit Fund (MCF) or PharmAccess ever spoken to you about their program?
   1. If no: Have you ever heard of the MCF/PharmAccess loan program?
   2. If still no, go to question 23
10. What are the benefits of the MCF/PharmAccess loan program?
11. What are the challenges of the MCF/PharmAccess loan program?
12. Do you have a loan from PharmAccess/MCF or have you applied for a loan from PharmAccess/MCF?
13. If did not take a loan or apply for a loan: Why weren’t you interested in a loan?
14. If took a loan or applied for a loan with MCF: Why did you decide to take a loan?
    1. What was the process for obtaining the loan?
    2. What were the conditions of the loan? *Probe:* amount, repayment period, collateral, others
    3. What are you using the loan for?
    4. Why is this important to your clinic?
    5. Do you think you will have accomplished everything you planned by the time the repayment period is over? Why?
    6. Do you expect to take another loan after you finish repaying the current one? Why?
15. How do you think the MCF program compares to your loan options from a regular bank? Why?
16. Have you ever taken a loan for your clinic before, not as part of MCF/PharmAccess?
    1. If yes: Where did you get the loan from?
    2. What did you use it for?
    3. Why was this important to your clinic?
    4. What were the benefits of having that loan?
    5. What were the challenges?
17. What do you know about the relationship between MCF, SafeCare and BlueStar?

**III. NHIS**

1. Now I’d like to talk about NHIS. Are many people in this area enrolled with NHIS?
   1. How many of your clients are enrolled with NHIS? (*Probe:* how many out of 10 have NHIS?)
2. What are the requirements for a facility to become accredited with NHIS?
   1. Which of these requirements were/would be most difficult for your facility to meet? Why?
   2. What kind of assistance could/would have helped you gain accreditation more easily?
   3. How could BlueStar help you to gain accreditation?
   4. How could PharmAccess, or MCF or SafeCare, help you to gain accreditation?
3. Has the facility ever applied for NHIS accreditation or has it ever been accredited?
4. If no: Why have you not applied for accreditation?
   1. What kind of information/assistance would you need to apply?

1. If yes : Why did you decide to apply for accreditation?
   1. Can you describe the process to become accredited?
   2. What were the challenges you faced during this process?
   3. What kind of assistance might have helped you with these challenges?
   4. If application was rejected/still pending: Why was your application not approved or why hasn’t it been approved yet?
      1. *Probes:* How have you followed up? What happened?
2. If facility is accredited: How to you find participation in NHIS?
   1. What are the benefits of participating?
   2. What are the challenges?
   3. How long does it take for your claims to be paid?
   4. How does this affect your facility?
   5. How do you cover the expenses while you wait for your claims to be paid?
   6. If you have any challenges with NHIS, how do you resolve them?
   7. How has joining NHIS affected your facility finances?
   8. Do you have the biometric card system for NHIS?
      1. If yes: What are the benefits of this system?
      2. What are the challenges?
3. Is there anything else you would like to tell me about your experience with NHIS?

**Demographic Sheet: AHME Provider Round 2**

**Session ID:** [ ] [ ] [ ] **Date (DD/MM/YY)**: ____ /____ / ____

| No. | Question | Coding Categories |
| --- | --- | --- |
| 1 | Gender | 🞏 1. Male |
|  |  | 🞏 2. Female |
| 2 | How old are you (in years)? | \|___\|___\| |
| 3 | What is the highest level of education you have completed? | 🞏 1. Never went to school or less than completing primary |
|  |  | 🞏 2. Primary education |
|  |  | 🞏 3. Secondary education |
|  |  | 🞏 4. Vocational/trade school |
|  |  | 🞏 5. University/other tertiary |
|  |  | 🞏 6. Masters/doctorate |
| 4 | What is your technical qualification? | 🞏 1. Medical doctor |
|  |  | 🞏 2. Medical assistant |
|  |  | 🞏 3. Nurse |
|  |  | 🞏 4. Community health/auxiliary nurse |
|  |  | 🞏 5. Midwife |
|  |  | 🞏 6. Health assistant |
|  |  | 🞏 7. Clinical Officer |
|  |  | 🞏 9. Other 🡪 please specify ______________________________ |
| 5 | Are you the owner of this facility? | 🞏 0. No |
|  |  | 🞏 1. Yes |
| 6 | What is your position title? | ______________________________ |
| 7 | How long have you been practicing (in years)? *(N/A if admin with no medical degree)* | \|___\|___\| |
| 8 | How long have you been working at this facility (in years)? | \|___\|___\| |
| 9 | What type of facility is this? | 🞏 1. Hospital |
|  |  | 🞏 2. Health center |
|  |  | 🞏 3. Clinic |
|  |  | 🞏 4. Maternity home |
|  |  | 🞏 5. Dispensary |
| 10 | What year did you join NHIS? *(N/A if not accredited)* | __________________________________ |
| 11 | How many medical staff does this facility have? (doctors, nurses, midwives, medical assistants) | Full Time Locum  \|___\|___\| \|___\|___\| |
| 12 | How many non-medical (support) staff does this facility have? (cleaners, security) | \|___\|___\| |
| 13 | What health services are offered at this facility? *(check all that are applicable)* | 🞏 1. Family planning |
|  |  | 🞏 2. Antenatal/ANC |
|  |  | 🞏3. Delivery |
|  |  | 🞏 4. Postnatal care/PNC |
|  |  | 🞏 5. Post abortion care |
|  |  | 🞏 6. STI |
|  |  | 🞏7. HIV counseling, testing & management |
|  |  | 🞏 8. Malaria |
|  |  | 🞏 9. Tuberculosis (TB) |
|  |  | 🞏 10. Diarrhea |
|  |  | 🞏 11. Respiratory tract infections |
|  |  | 🞏 12. Nutrition |
|  |  | 🞏 13. Immunizations |
|  |  | 🞏 13. Cervical cancer screening |
|  |  | 🞏 14. Other 🡪 please specify ______________________________ |
