# Supplementary material for: Private healthcare provider experiences with social health insurance schemes: Findings from a qualitative study in Ghana and Kenya
Source: PLoS One. 2018 Feb 22;13(2):e0192973. doi: 10.1371/journal.pone.0192973 (PMC5823407; doi:10.1371/journal.pone.0192973)
Supplement: S3 Text — Kenya verbal consent form. (DOCX) [file pone.0192973.s003.docx]

UNIVERSITY OF CALIFORNIA, SAN FRANCISCO

VERBAL INFORMED CONSENT TO PARTICIPATE IN A RESEARCH STUDY

IN-DEPTH INTERVIEWS

**Study Title:** Qualitative process evaluation of the African Health Markets for Equity (AHME) partnership

**IRB No**: 13-11045

**Version Number and Date**: Version 1.0, 14 JUN 2013

Thank you for agreeing to talk with us today. We are from the University of California, San Francisco in the United States of America and from Innovations for Poverty Action Kenya. We would like to tell you about your potential part in the study.

**Purpose**

This is a research study about the African Health Markets for Equity (AHME) partnership, a group of organizations that provide private health care and technology services. You are being asked to take part in this study because you are either a patient or healthcare provider within an AHME study area.

Your participation in this research study is voluntary. Please take your time to make your decision. If you have any questions you can ask me at anytime.

**What will happen if I take part in this study?**

If you agree to participate in this research study, you will be asked to have a one-on-one interview for an informal discussion about your attitudes towards health services and your experience with the AHME network. One to two researchers will lead the interview. We will ask you some basic questions about your attitudes towards health issues and your experiences with the AHME services and franchise networks. With your permission, we will tape-record the discussion. The interview will last about one to one and a half hours.

**Can I stop being in the study?**

Yes. You can stop participating in the study at any time. If you decide to participate, you may refuse to answer any question that you do not want to answer.

**Are there risks to participating in the study?**

There are no major risks. Some of the questions asked during the interview may make you feel uncomfortable or ask about your personal attitudes. You may refuse to answer any question you do not want to answer. You may stop the discussion at any time.

**Are there benefits to participating in the study?**

There is no direct benefit to you from participating in this study. The information that you provide will help us to understand the health services and franchise networks in your community. You will not be paid for taking part in this study.

**Do I have to participate?**

No. You may choose not to participate in the study. There are no penalties to you if you choose not to participate.

**Will information about me be kept private?**

The information you share today will be kept private. We will not share anything you tell us with anyone outside of the study team. No information that can identify you, such as your name or date of birth, will be written down. Tapes and interview notes will only contain number identification, and will be kept in a locked cabinet at the Innovations for Poverty Action Kenya offices. Only research staff have access to the cabinets and all tapes and interview notes will be destroyed at the end of the study.

**Who can answer my questions about the study?**

If you have any questions, concerns, or complaints about this study, you may contact Suleiman Asman, Research Manager for Innovations for Poverty Action-Kenya at 0708233867. If you have any further questions about your rights as a research participant, please contact Kenya Medical Research Institute (KEMRI):

PO Box 54840-0020, Nairobi

Telephone numbers 020-2722541, 0722205901, 0733400003

Email: erc@kemri.org

You will receive a copy of this form for you to keep if you have any further questions.

**Oral consent:**

1. Do you have any questions about the research or anything I just said?
2. If you think I have answered all your questions about this study, please tell me, do you agree to participate?

🞏 YES 🡪 *RESEARCHER SIGN STATEMENT BELOW*

🞏 NO 🡪 *STOP AND THANK INDIVIDUAL*

1. By signing below, I confirm that the individual has given consent freely.

**Researcher Signature**:_________________________________________
