# Supplementary material for: Private healthcare provider experiences with social health insurance schemes: Findings from a qualitative study in Ghana and Kenya
Source: PLoS One. 2018 Feb 22;13(2):e0192973. doi: 10.1371/journal.pone.0192973 (PMC5823407; doi:10.1371/journal.pone.0192973)
Supplement: S4 Text — Ghana written consent form. (DOCX) [file pone.0192973.s004.docx]

Study Title: Qualitative process evaluation of the African Health Markets for Equity (AHME) partnership

IRB No: 13-11045

Version Number and Date: 5 October 2015

Thank you for agreeing to talk with us today. My name is [INTERVIEWER 1 NAME] and this is [INTERVIEWER 2 NAME - *if applicable*]. We are from the University of California, San Francisco in the United States of America and from Innovations for Poverty Action Ghana. We would like to tell you about your potential part in the study.

Purpose

This is a research study about the African Health Markets for Equity (AHME) partnership, a group of organizations that provide private health care and technology services. You are being asked to take part in this study because you are either a patient or healthcare provider within an AHME study area, or you are a key member of the AHME partnership. Approximately 300 people will participate in this study over the course of four years from 2013 - 2017. The Principle Investigator for this study is Dominic Montagu at the University of California in San Francisco in the United States of America.

Your participation in this research study is voluntary. Please take your time to make your decision. If you have any questions you can ask me at any time.

What will happen if I take part in this study?

If you agree to participate in this research study, you will be asked to have a one-on-one interview for an informal discussion about your attitudes towards health care, your experience with the AHME program and your perception of health services and topics. One to two researchers will lead the interview. With your permission, we will tape-record the discussion. The interview will last about one hour.

Can I stop being in the study?

Yes. You can stop participating in the study at any time. If you decide to participate, you may refuse to answer any question that you do not want to answer.

Are there risks to participating in the study?

There are no major risks. Some of the questions asked during the interview may make you feel uncomfortable or ask about your personal experience and attitudes. You may refuse to answer any question you do not want to answer. You may stop the discussion at any time.

Are there benefits to participating in the study?

There is no direct benefit to you from participating in this study. The information that you provide will help us to understand the impact of the AHME services for the communities that the program serves and how the AHME partnership works. The results of the study will be regularly presented to the AHME partners in order to help them improve the program. You will not be paid for taking part in this study.

Do I have to participate?

No. You may choose not to participate in the study. There are no penalties to you if you choose not to participate.

Will information about me be kept private?

We will do our best to make sure that your personal information is kept private. We will keep the information you tell us in the interview confidential, and we will not share your personal information with anyone outside of the research team. We will not collect anything that will identify you. Tapes and interview notes will only contain number identification. All tapes and interview notes will be kept in a locked cabinet. At the end of the study when all the data has been analyzed, all study documents including tapes and interview notes will be destroyed. Please remember that we want to have an open conversation. There are no right answers.

Who can answer my questions about the study?

If you have any questions, concerns, or complaints about this study, you may contact Abigail Amartey at Innovations for Poverty Action-Ghana at +233 302 790 372. If you have any further questions about your rights as a research participant, please contact ERC Administrator on Telephone number 024-4712919

Email: nanatuesdaykad@yahoo.com

In Kenya, you may contact **Cynthia Onyango**, Research **Associate** for Innovations for Poverty Action-Kenya at **+254 707 387 429**. If you have any further questions about your rights as a research participant, please contact Kenya Medical Research Institute (KEMRI):

PO Box 54840-0020, Nairobi

Telephone numbers 020-2722541, 0722205901, 0733400003

Email: erc@kemri.org

Consent and copy of the form

Your participation in the study is voluntary. You may choose not to be in the study, or to leave the study at any time. You will receive a copy of this form for you to keep if you have any further questions.

Informed consent:

I have received an explanation of the study. I have had the opportunity to ask questions and any questions I have asked have been answered to my satisfaction. I consent voluntarily to participate in this research and understand that I have the right to withdraw from the study at any time without this affecting my medical care or participation in the AHME program.

[If interview is being conducted by phone, turn on the tape recorder, read the informed consent paragraph, ask participant “Do you agree to this statement”, and have the participant state their name and today’s date.]

Printed Name

Signature

Date (day/month/year)

If illiterate

I have witnessed the accurate reading of the consent form to the potential participant and the individual has had the opportunity to ask questions. I confirm that the individual has given consent freely.

__________________________________________________________ AND Thumb print of participant

|  |
| --- |

Printed Name of Witness

________________________________________________________

Signature of Witness

__________________________________________________________

Date (day/month/year)

---------------------------------------------------------------------------------------------------------------------

Before we begin the interview, I would like to get your permission to tape record the interview. The tapes will only be available to the research team. Are you willing to allow this interview to be recorded? Do I have your consent to participate in the study?

[If participant says yes, CONTINUE]
