# Supplementary material for: Private healthcare provider experiences with social health insurance schemes: Findings from a qualitative study in Ghana and Kenya
Source: PLoS One. 2018 Feb 22;13(2):e0192973. doi: 10.1371/journal.pone.0192973 (PMC5823407; doi:10.1371/journal.pone.0192973)
Supplement: S5 Text — Codebook. (DOCX) [file pone.0192973.s005.docx]

| **CODE FAMILY: NHIF** | | |
| --- | --- | --- |
| **Accreditation assistance** | | Discussion of the assistance the provider needs or would like to receive to become accredited with NHIF |
|  | - assessment of institution | Need assistance/knowledge of the assessment process |
|  | - better coordination with other registration | The provider thinks the accreditation process should be more coordinated with other licensing/registration requirements they must fulfill |
|  | - continuous monitoring | Provider wants continuous quality monitoring to help them maintain NHIF standards |
|  | - financial | General financial assistance needed and/or funds to make specific improvements |
|  | - franchisor | How the franchisor could provide support (ex. Writing a letter to NHIF as a partner) |
|  | - help to improve standards | Assistance improving overall quality/services of facility |
|  | - info on costs | Information on costs of accreditation |
|  | - info/guidance on process | Information on how to go about the accreditation process |
|  | - info/assistance from NHIF | Info/assistance directly from NHIF (no mention of franchise, MCF/SafeCare) |
|  | - intermediary to deal with system | Provider thinks it would be useful for an organization to help link them to NHIF |
|  | - none | Does not need any assistance to get accreditation |
| **Accreditation payment** | | For discussion of the costs (official or otherwise) of accreditation |
| **Accreditation process** | | Discussion about the accreditation process (for accreditation requirements see “NHIF requirements” below). |
|  | - accreditation level | Discussion of differences in class at which facility is accredited (B, C etc.) |
|  | - changes made to clinic | Changes the provider made to the facility to meet NHIF requirements |
|  | - general | General information regarding the process for being accredited |
|  | **-** time – general | Inpatient/outpatient not specified |
|  | - time – inpatient | Time taken to get inpatient accreditation |
|  | - time – outpatient | Time taken to get outpatient accreditation |
|  | - waiting inspection | Facility is waiting for NHIF inspectors to move forward with accreditation process |
|  | - why not approved | Reasons for rejection for those who applied for accreditation and were not accepted |
| **Client concerns about NHIF** | | Discussion of problems/concerns that clients have about NHIF |
| **Complain to NHIF** | | Options for discussing problems/complaints with NHIF |
|  | - branch/office | Go to the branch headquarters to talk to staff |
|  | - opt out | Can just leave the NHIF system |
|  | - nothing to do | There is nothing they can do to make NHIF address challenges |
|  | - reps who come to clinic | There are NHIF representatives who come to the facility |
| **Considered accreditation** | | The facility has considered accreditation/is potentially interested but not applied |
|  | - did not follow up | The provider has not made any effort to follow up on accreditation process |
|  | - general | Interested in accreditation but not clear if inpatient or outpatient |
|  | - never | Have not considered getting accreditation |
|  | - inpatient | For the inpatient scheme |
|  | - outpatient | For the outpatient scheme |
| **Do not treat NHIF patients differently** | | The provider says he does not treat NHIF card clients differently from paying clients |
| **Maintaining NHIF accreditation** | | Any discussion of how NHIF accreditation is maintained after initial approval |
| **Need for client awareness of NHIF** | | Providers sees that community does not have good awareness of NHIF |
| **NHIF benefits** | | Discussion of benefits (experienced or perceived) from participation in NHIF. Double-code with “NHIF INPATIENT” or “NHIF OUTPATIENT” if is specific to one scheme. |
|  | - allows people to pay | NHIF benefit to clients in that they can access services that would not be available to them due to cost |
|  | - clients attached to facility | NHIF (outpatient) means that clients are registered at facility so they keep coming back, is no competition for them |
|  | - consistent cash flow | NHIF pays regularly, unlike patients who may take a long time to settle their bills |
|  | - higher standards in clinic | Being part of NHIF encourages/requires facility to maintain higher standards |
|  | - increased client flows | Increases number of clients coming to facility |
|  | - increased client confidence/satisfaction | Clients have more confidence and trust in the clinic since they see it as an affiliation to the government, are happier with facility. |
|  | - money paid as a lump sum | NHIF pays money as a lump sum over a period of time |
|  | - increased income | The facility makes money from participation in NHIF |
|  | - reaches more people | The clinic can reach more clients who have insurance |
| **NHIF challenges** | | Discussion of challenges (experienced or perceived) of participation in NHIF. Double-code with “NHIF INPATIENT” or “NHIF OUTPATIENT” if is specific to one scheme. |
|  | - clients don’t understand how it works | Clients want services that aren’t covered, don’t understand capitation etc. |
|  | - corruption | Any complaint of unfair treatment/demanding of bribes etc. from NHIF (always ALSO code “NHIF perception of bias/lack of consistency in standards”) |
|  | - crowding/long patient waits | The clients are too many for what the facility can accommodate |
|  | - delay in reimbursement/payment | Delay in getting reimbursements/payments/claims paid by NHIF |
|  | **-** getting accreditation level changed | Process for getting facility accredited at a higher level (with higher reimbursements) |
|  | **-** getting enough clients under capitation | Having enough clients register under outpatient that capitation rates are ok financially |
|  | **-** Inconsistent information/application of rules | Get different information about what the facility should be doing, rules, coverage etc. |
|  | **-** length of time | The delays in getting accredited |
|  | - limited services covered | NHIF does not cover sufficient/all services, or only covers select services |
|  | - low reimbursement/capitation | Provider is not reimbursed sufficiently for services; capitation rate is too low |
|  | - meeting requirements | Worried they will not be able to meet requirements |
|  | - none | There are no challenges |
|  | - offering all services for outpatient | Difficult for facility to offer all the services they want under outpatient, e.g. dental, optical |
|  | - paperwork | Paperwork required for NHIF (incl. reimbursements) |
|  | - poor communication | Lack of communication/notification or response from NHIF when provider experiences problems |
|  | - referral/continuity of care | Concerns about NHIF recommendations about referring clients (e.g. under capitation) |
| **NHIF client enrollment requirements/process** | | Process or requirement from the client side for enrollment in NHIF |
| **NHIF common in community?** | | Code all answers to the question about how common NHIF is among the community/clients |
| **NHIF INPATIENT** | | Use only to double-code with other NHIF codes when specific to inpatient |
| **NHIF OUTPATIENT** | | Use only to double-code with other NHIF codes when specific to outpatient |
| **NHIF perception of bias/lack of consistency in standards** | | Any discussion of bias, corruption or inconsistent application of accreditation standards by NHIF |
| **NHIF requirements** | | For discussion of the requirements for NHIF accreditation. |
|  | - clinic registration | The clinic as a business must be registered as a limited company and not a sole proprietorship |
|  | - difficult to meet | Requirements are difficult, or aspects of requirements that are most difficult |
|  | - don't know | Do not know/understand requirements |
|  | - general | Discussing general requirements of getting accredited |
|  | - inpatient | Specific discussion of inpatient requirements |
|  | - outpatient | Specific discussion of outpatient requirements |
| **Reimbursement process** | | Discussion of the reimbursement process (that does not count as a challenge) |
| **Understanding of how NHIF works** | | Any discussion of how NHIF functions that does not fit well into other codes |
| **Why apply NHIF** | | Reasons for applying for/wanting to apply for NHIF accreditation. Double-code with “NHIF INPATIENT” or “NHIF OUTPATIENT” if is specific to one scheme. |
|  | - competition from other facilities | Would/were losing clients to other facilities that accept NHIF |
|  | - customer demand | Clients ask if facility will accept NHIF |
|  | - financial | It is profitable for the facility to be part of NHIF or provides revenue to facility |
|  | - franchise request | The provider will apply for NHIF if the franchisor tells them to apply |
|  | - gives clients confidence | Clients will believe the facility is good quality if they see it accredited with NHIF |
|  | - government push | Government is promoting and/or requiring NHIF (according to provider) |
|  | - private insurance prefer facilities with accreditation | Helps the facility be attractive to private insurance companies |
|  | - seen other facilities enroll | Know other facilities that joined, and wanted to follow |
|  | - so clients can pay | Felt clients weren’t able to pay for services, or weren’t settling bills, so wanted them to be able to pay with card |
| **Why not applied NHIF** | | Reasons for not applying for/wanting to apply for NHIF accreditation. Double-code with “NHIF INPATIENT” or “NHIF OUTPATIENT” if is specific to one scheme. |
|  | - cannot meet requirements | Clinician does not feel he/she can meet the requirements to become accredited |
|  | - challenges with scheme | Did not apply (for additional scheme) due to challenges with scheme they are already in |
|  | - corruption/lack of trust | Provider does not trust the system |
|  | - cumbersome procedure | Accreditation process is difficult, arbitrary etc. |
|  | - don't have inpatient services | Does not have inpatient services (or “beds”) |
|  | - experience of other facilities | Negative experiences of other facilities/friends who have applied |
|  | - few clients enrolled | Does not think that facility’s clients are enrolled with NHIF |
|  | - lack of capacity | Don’t have space to attend to additional clients |
|  | - middle class clientele | Want to remain attractive and open for middle/upper class clientele |
|  | - not enough information | Lacking information on the process |
|  | - not fulltime provider | The provider works in the clinic on a part time basis and therefore would not be available to patients at all times. |
| **CODE FAMILY: NHIS GHANA** | | |
| **Accreditation assistance** | | Discussion of the assistance the provider needs or would like to receive to become accredited with NHIS |
|  | - assessment of institution | Need assistance/knowledge of the assessment process |
|  | - better coordination with other registration | The provider thinks the accreditation process should be more coordinated with other licensing/registration requirements they must fulfill |
|  | - continuous monitoring | The provider want continuous quality monitoring to help them maintain NHIS standards |
|  | - financial | General financial assistance needed and/or funds to make specific improvements |
|  | - franchisor | How the franchisor could provide support (ex. writing a letter to NHIS as a partner) |
|  | - help to improve standards | Assistance improving overall quality/services of facility |
|  | - info on costs | Information on costs of accreditation |
|  | - info/guidance on process | Information on how to go about the accreditation process |
|  | - info/assistance from NHIF | Info/assistance directly from NHIS (no mention of franchise, MCF/SafeCare) |
|  | - intermediary to deal with system | Provider thinks it would be useful for an organization to help link them to NHIS |
|  | - none | Does not need any assistance to get accreditation |
| **Assistance with NHIS generally** | | For discussion of any assistance the provider wants in dealing with NHIS that is not related to the accreditation process |
|  | - provide loans/financial assistance | Wants loans/financial support to cover gaps when NHIS has not paid |
| **Biometric system** | | For any discussion of the NHIS biometric card system |
|  | - electricity problems | Trouble running the biometric system on the generator |
|  | - helps identify clients | Is useful to identify clients correctly |
|  | - helps reduce human resource load | Less workers needed for verification of clients if the machine is there |
|  | - not using yet | The clinic is not set up with a biometric system yet |
|  | - only some patients use it | Only some patient use the biometric machine others use the old card |
|  | - sometimes doesn’t recognize client | Sometimes doesn’t recognize the client’s card or fingerprint |
| **Complain to NHIS** | | Options for discussing problems/complaints with NHIS |
|  | - branch/office | Go to the branch headquarters to talk to staff |
|  | - nothing to do | Is nothing they can do to make NHIS address challenges |
| **Effects of delayed payments** | | Discussion of how delay in payments from NHIS affects the facility, or how the provider deals with these effects |
|  | **-** challenge making improvements | Difficult to improve facility when payment is held up for months |
|  | - challenges to pay bills | It is difficult for the provider to pay regular operating costs of the facility |
|  | - challenges to pay employees | It is difficult for the provider to pay staff |
|  | - challenges to stock drugs | The provider cannot stock as many drugs due to cash shortages |
|  | - facilities leaving NHIS | Some facilities are deciding to withdraw from the NHIS system |
|  | - lose staff | The provider has lost staff because s/he couldn’t pay them on time |
|  | - rely on cash paying clients | The facility relies on cash-paying clients to keep some money coming in while waiting for payments |
|  | - take loan | The provider has taken a loan to cover operating cost shortages |
|  | - use personal money to cover costs | The provider uses their personal money to cover operating cost shortages |
| **NHIS accreditation process** | | Discussion of the accreditation process for NHIS |
|  | - accreditation level | The level at which the facility is accredited (which affects the services that are covered by NHIS, and reimbursement rates) |
|  | - corruption | Any perception/experience of corruption in the process |
|  | - general | General discussion of how the process works |
|  | - lack of information | Provider lacks/lacked information on accreditation process |
|  | - disorganized | The process to become accredited is unorganized |
|  | - time | The time it took to become accredited |
| **NHIS benefits** | | Discussion of benefits (experienced or perceived) from participation in NHIS |
|  | - clients come for care more regularly | Clients do not wait so long to come for care because it is covered |
|  | - consistent cash flow | Consistent cash flow due to capitation |
|  | - good for community | Accepting NHIS allows members of the community to receive care who might not have been able to receive care without it |
|  | - more clients | The facility gets more clients |
| **NHIS challenges** | | Discussion of challenges (experienced or perceived) of participation in NHIS |
|  | - clients have to copay | Clients have to copay for services (incl. financial burden and that clients don’t like/understand copays) |
|  | - deductions from claims | NHIS makes deductions from the claims that the provider files, and doesn’t pay for these things |
|  | - delay in payments | Payments/reimbursement of claims from NHIS are delayed |
|  | - financial | Providers can make more for specific services when they are not part of NHIS than they can when they are accredited |
|  | - lack of frequent reviews of the price lists | NHIS does not review the medicine price list and this affects the providers as the drugs prices keep going up in the market |
|  | - patients move around | Patients move around to different clinics often, so hard to follow-up and monitor |
|  | - poor communication of policy changes | NHIS does not communicate well to providers or clients when policies or services covered are changed |
|  | - reporting | The reporting required is very tedious |
|  | - restrictions on services/meds covered | Limits on the services or drugs that are covered under NHIS (including if this is related to the level of accreditation that the facility has) |
|  | - space | The clinic was too small for accreditation at first, needed to expand to be eligible |
| **NHIS common in community?** | | Code all answers to the question about how common NHIS is among the community/clients |
| **NHIF perception of bias/lack of consistency in standards** | | Any discussion of bias, corruption or inconsistent application of accreditation standards by NHIS |
| **NHIS requirements** | | Discussion of the requirements to be accredited by NHIS |
|  | - difficult to meet | The requirements that were difficult for the provider to meet |
|  | - don’t know | The provider does not know the requirements for becoming accredited |
|  | - general | General discussion of the requirements |
|  | - no challenges | The requirements are not difficult to meet |
| **Resolve NHIS challenges** | | Discussion of how the providers deals with/resolves challenges with NHIS |
|  | - go to office | Go to the office to talk with staff |
|  | - need to listen to providers more | Provider expresses that NHIS should involve/listen to providers more in making their policies, or running the scheme |
|  | - write letter | Provider writes a letter to NHIS about problem |
| **Why apply NHIS** | | Reasons for applying for/wanting to apply for NHIS accreditation |
|  | - competition from other facilities | Would/were losing clients to other facilities that accept NHIS |
|  | - customer demand | Clients ask if facility will accept NHIS |
|  | - financial | It is profitable for the facility to be part of NHIS or provides revenue to facility |
|  | - franchise request | The provider will apply for NHIS if the franchisor tells them to apply |
|  | - gives clients confidence | Clients will believe the facility is good quality if they see it accredited with NHIS |
|  | - good for community | Belonging to NHIS is good for the community |
|  | - government push | Government is promoting and/or requiring NHIS (according to provider) |
|  | - help grow business | By joining NHIS will help grow the clinic |
|  | - private insurance prefer facilities with accreditation | Helps the facility be attractive to private insurance companies |
|  | - seen other facilities enroll | Know other facilities that joined, and wanted to follow |
|  | - so clients can pay | Felt clients weren’t able to pay for services, or weren’t settling bills, so wanted them to be able to pay with card |
| **Why not applied NHIS** | | Reasons for not applying for/wanting to apply for NHIS accreditation. |
|  | - cannot meet requirements | Clinician does not feel he/she can meet the requirements to become accredited |
|  | - challenges with scheme | Did not apply (for additional scheme) due to challenges with scheme they are already in |
|  | - corruption/lack of trust | Provider does not trust the system |
|  | - cumbersome procedure | Accreditation process is difficult, arbitrary etc. |
|  | - don't have inpatient services | Does not have inpatient services (or “beds”) |
|  | - experience of other facilities | Negative experiences of other facilities/friends who have applied |
|  | - few clients enrolled | Does not think that facility’s clients are enrolled with NHIS |
|  | - lack of capacity | Don’t have space to attend to additional clients |
|  | - not enough information | Lacking information on the process |
|  | - not fulltime provider | The provider works in the clinic on a part time basis and therefore would not be available to patients at all times. |
|  | - not ready yet | The clinic is making changes and not ready to have NHIS come in and evaluate them |
